# Population genetics and host specificity of *Varroa destructor* mites infesting eastern and western honeybees

**DOI:** 10.1007/s10340-020-01322-7

**Published:** 2021-01-15

**Authors:** Zheguang Lin, Shuai Wang, Peter Neumann, Gongwen Chen, Paul Page, Li Li, Fuliang Hu, Huoqing Zheng, Vincent Dietemann

**Affiliations:** 1 College of Animal Sciences, Zhejiang University, Hangzhou, China; 2 College of Animal Science and Technology, Yangzhou University, Yangzhou, China; 3 Swiss Bee Research Center, Agroscope, Bern, Switzerland; 4 Institute of Bee Health, Vetsuisse Faculty, University of Bern, Bern, Switzerland; 5 Department of Ecology and Evolution, University of Lausanne, Lausanne, Switzerland

**Keywords:** Host shift, Gene flow, Genetic diversity, Host specificity, Population genetics, Dispersal, Honeybee, Parasitic mite, *Varroa destructor*

## Abstract

In a globalized world, parasites are often brought in contact with new potential hosts. When parasites successfully shift host, severe diseases can emerge at a large cost to society. However, the evolutionary processes leading to successful shifts are rarely understood, hindering risk assessment, prevention, or mitigation of their effects. Here, we screened populations of *Varroa destructor*, an ectoparasitic mite of the honeybee genus *Apis*, to investigate their genetic structure and reproductive potential on new and original hosts. From the patterns identified, we deduce the factors that influenced the macro- and microevolutionary processes that led to the structure observed. Among the mite variants identified, we found two genetically similar populations that differed in their reproductive abilities and thus in their host specificity. These lineages could interbreed, which represents a threat due to the possible increased virulence of the parasite on its original host. However, interbreeding was unidirectional from the host-shifted to the nonshifted native mites and could thus lead to speciation of the former. The results improve our understanding of the processes affecting the population structure and evolution of this economically important mite genus and suggest that introgression between shifted and nonshifted lineages may endanger the original host.

**Supplementary Information:**

The online version contains supplementary material available at 10.1007/s10340-020-01322-7.

## Key Message

- Knowledge of the relationship between the ectoparasitic mite *Varroa destructor* and its two honeybee host species is lacking.
- We took advantage of the sympatric occurrence of the host-shifted mite haplotype in Chinese populations of both original and novel hosts.
- A genetically distinct mite population to the host-shifted lineage was identified in the original host.
- Unidirectional gene flow occurred between the lineages, potentially leading to speciation on the new host and to damage to the original host.

## Introduction

Emerging infectious diseases present a major challenge to the health of humans and our domesticated animal and crop species (Daszak et al. 2000; Morens et al. 2004). Such diseases are often the product of a host shift, in which a pathogen broadens its host spectrum by successfully using a new host species (Woolhouse et al. 2005). Despite a high number of host shifts reported in the literature, the evolutionary processes that favor host shifts are poorly understood. Unveiling the factors driving these processes is crucial to our ability to predict and mitigate their effects, as well as potentially preventing their occurrence (Dietemann et al. 2012; Huyse et al. 2005; Poullain and Nuismer 2012; Woolhouse et al. 2005). The evolutionary potential of parasites—defined as their ability to overcome resistance mechanisms in the host (McDonald and Linde 2002) or to shift host—is affected by factors such as dispersal patterns, reproductive traits, gene flow, and host specificity (Huyse et al. 2005). To investigate these factors, we used the ectoparasitic mite, *Varroa destructor*, which is native to Asia and infests an economically important insect, the Western honeybee, *Apis mellifera*.

In the middle of the nineteenth century, the Western honeybee, *A. mellifera*, was brought into contact with its sister species, the Eastern honeybee, *Apis cerana* (Chantawannakul and Ramsey 2018). This sympatry gave *V. destructor*, which originally infested *A. cerana*, the opportunity to enter colonies of the imported Western honeybee. The Korea 1-1 (K1-1) variant of *V. destructor* successfully infested and reproduced in *A. mellifera* colonies and spread worldwide thanks to the global honeybee trade (Anderson and Trueman 2000; Solignac et al. 2005; Traynor et al. 2020). Whereas *V.* *destructor* only reproduces on drone brood of their original host, the host-shifted lineage has the ability to reproduce on both worker and drone brood of the new host, which results in an exponential propagation of the mite with a negative impact on colony health. Because of its global distribution and detrimental effects on the host (Anderson and Trueman 2000; Anderson 2000; Carreck and Neumann 2010; Solignac et al. 2005), the invasive lineage is considered to be a major biotic threat to the survival of managed and wild *A.* *mellifera* populations and to the ecological services they deliver (Dietemann et al. 2012; Evans 2019; Jaffé et al. 2010; Nazzi and Le Conte 2016; Pirk et al. 2017; Rosenkranz et al. 2010).

Despite this negative impact fueling numerous studies of this parasite, little attention has been devoted to the evolutionary processes of *Varroa* spp. in their original distribution range and host (Dietemann et al. 2012). In particular, no study to date has targeted an *A. cerana* host population infested by the K1-1 variant of *V. destructor*, from which insights into the evolutionary processes that led to the host shift by this invader can be gained. To fill this gap, we screened *A. cerana* colonies for mites in eastern China, where the K1 haplogroup of *V. destructor* has been previously found (Navajas et al. 2010). To better define the distribution range and genetic diversity of this haplogroup and to account for variations in host parasite coevolution scenarios (Thompson 2005), we sampled over a wide area of eastern China.

In order to identify the genetic diversity, host specificity, and gene flow involved in the micro- and macroevolutionary processes underlying host–parasite relationships (Huyse et al. 2005), we combined experimental infestations with tools from population genetics (Criscione et al. 2005; De Meeûs et al. 2007; Wilson et al. 2005). We sampled several mites per host colony from several colonies per location to assess mite dispersal and drifting between host species. In addition to using mitochondrial DNA typing to identify mite haplogroups and variants and to measure their genetic diversity, we used nuclear DNA analysis. This technique provides information on paternal gene flow that is undetected in exclusive analysis of mitochondrial DNA and provides a more comprehensive mapping of population structure and dynamics, as well as a more detailed description of reproduction systems (De Meeûs et al. 2007; Dietemann et al. 2019; Hodel et al. 2016).

Our findings show that, in contrast to nonshifted *V. destructor* variants in eastern China, the host-shifted and invasive lineage can reproduce on worker and drone brood of both original and new host species. These different reproductive abilities and host specificity could explain the unidirectional gene flow observed from the shifted to the nonshifted populations. This gene flow could lead to speciation of the host-shifted lineage and to hybrid mites disrupting the equilibrium within the coevolved interaction, thereby threatening the original host. These results contribute to a more precise understanding of the factors and evolutionary processes that have shaped the *V. destructor* population structure and of the risk posed by the host-shifted lineage to the original host.

## Materials and methods

### Sampling

**Fig. 1:**
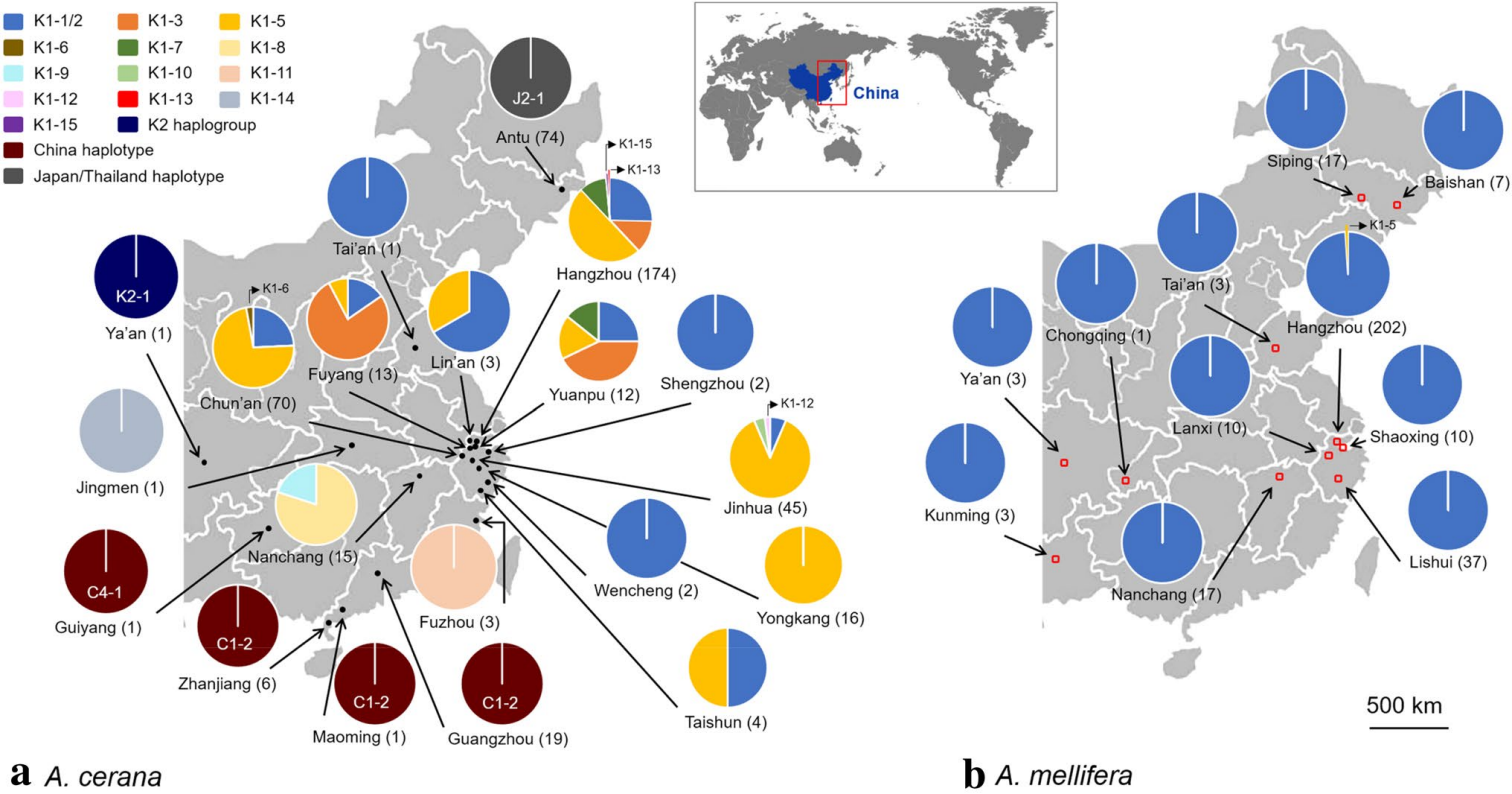
Maps of eastern China showing sampling localities, haplotypes, and variants of *Varroa destructor* mites infesting **a** *Apis cerana* (black dots) and **b** *Apis mellifera* (red hollow square). The numbers in parentheses following the locality names indicate sample size of mites sequenced. The haplotype, haplogroup and variant frequencies of the mites sampled at each locality are shown with pie charts

From 2013 to 2018, a total of 1149 adult female *V. destructor* mites were collected from capped brood cells, including 566 and 583 mites from 161 *A.* *cerana* colonies in 20 localities and 25 *A.* *mellifera* colonies in 11 localities in China (Fig. 1), respectively. Mite collection was performed following standard methods (Dietemann et al. 2013). Mites were collected from drone and worker brood in *A.* *mellifera* and *A. cerana* (with only a few mites found in the worker brood of the latter). These *V.* *destructor* samples were used for one or several of the following analyses/assays: mitochondrial and nuclear genotyping (Evans et al. 2013) and experimental infestations (Dietemann et al. 2013; Lin et al. 2018) (Fig. S1).

### Genotyping

#### DNA extraction

Total DNA of 463 *A. cerana* and 310 *A. mellifera* individual mites (Fig. S1) was extracted following the manufacturer’s instructions (DNeasy kit; Tiangen, Beijing, China). The quality and quantity of each DNA extract were then assessed using a Nanodrop Spectrophotometer (Thermo Fisher Scientific, Waltham, USA).

#### Mitochondrial DNA analyses

**Fig. 2:**
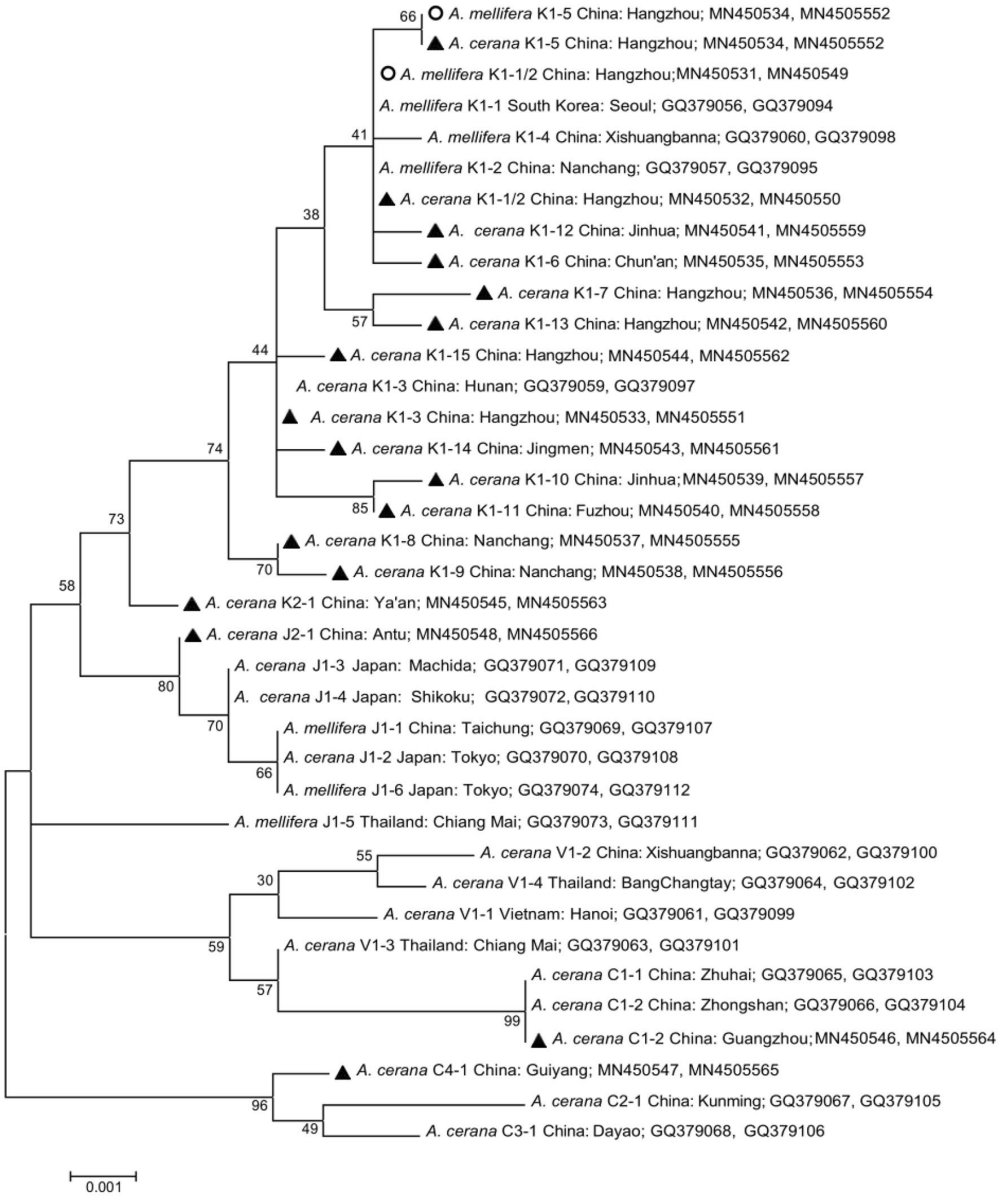
Neighbor-joining phylogenetic tree of *Varroa destructor* based on concatenated mtDNA sequences of the *cox1* and *cytb* genes. Symbols designate mites collected from *Apis* *mellifera* (empty circles) and *Apis cerana* (solid triangles) colonies, respectively, in this study. Host species and sampling localities (country and city) for one representative individual of each haplotype are indicated. All entries are followed by their GenBank accession numbers

An 821-bp fragment of the mitochondrial Cytochrome Oxidase I gene (*cox1*) and a 985-bp fragment of the cytochrome b gene (*cytb*) were amplified by PCR from individual mites (*N* = 463 from *A. cerana*, *N* = 310 from *A. mellifera*; Fig. S1) using published and novel primers (Table S1). PCR amplification was performed in a 50 μl reaction volume mixture (KOD FX Mix; Toyobo, Osaka, Japan); the profile consisted of an initial denaturation at 94 °C for 2 min, followed by 35 cycles at 94 °C for 30 s, annealing temperature Tm (see Table S1) for 30 s, 72 °C for 30 s, and a final elongation step at 72 °C for 5 min. Negative controls (water instead of DNA template) were included in each PCR run. Amplification efficiency was verified by electrophoresis of PCR products in 2.5% agarose gel stained with gelview (BioTeke, Beijing, China) and visualized under UV light. Positive PCR products were cleaned and sequenced in both directions (forward and reverse) by Sangon Biotech Co. Ltd. (Shanghai, China) on an ABI 3730xl automated DNA sequencer (Applied Biosystems, Foster City, USA). The sequences obtained were compared with the complete mitochondrial sequence of *V.* *destructor* (GenBank Accession Number: AJ493124.2; Navajas et al. 2002), as well as with various *Varroa* spp. haplotypes retrieved from the NCBI database (see Fig. 2). Sequences were edited in BioEdit V7.0.9.0 (Hall 1999).

To investigate the phylogenetic relationship between the samples, we performed an analysis of concatenated *cox1* and *cytb* gene sequences using the Neighbor-Joining algorithm. Sequence comparison between samples and building of the phylogenetic trees were conducted using MEGA6.0 (Tamura et al. 2013). The code K1-1/2 is used to designate K1-1 and K1-2 variants since their sequences are identical (Navajas et al. 2010). The sequences of one representative mite per variant were uploaded to GenBank with the accession numbers MN450531-MN450566.

#### Microsatellite analyses

A random sample of the haplotyped mites was also genotyped with six DNA microsatellites (Table S2) to consider the effect of paternal gene flow on population structure (*N* = 192 from *A.* *mellifera*, *N* = 339 from *A.* *cerana*; Fig. S1).

The forward primers were modified with fluorescent dyes (FAM, HEX, and TAMRA) in 5’-terminal by Sangon Biotech Co. Ltd. (Shanghai, China) to allow for allele detection. PCR temperature programs were similar to those used for haplotyping (see 2.2.2), except for Tm (Table S2). The PCR products were sequenced on an ABI 3730xl (internal standard: Liz500) by Sangon Biotech Co. Ltd. (Shanghai, China).

Microchecker was used to check for the occurrence of allele dropout. INEST was used to estimate null allele frequencies since it can account for the effects of inbreeding.

Bayesian clustering methods that are not reliant on a priori information (InStruct V1.0; Gao et al. 2007) were used to infer population structure and assess introgression levels within the mite samples (*N* = 531). The number of clusters in the dataset (*K*) varied from 1 to 25. InStruct was run with 10 simulations for each *K* value from 1 to 25, with 50,000 iterations for each simulation, following a burn-in period of 25,000 iterations using the admixture mode and correlated allele frequencies. The most likely number of clusters was then estimated using the method described by Evanno et al. (2005). DISTRUCT V1.1 (Rosenberg 2004) was used to graphically display the output files from InStruct.

To complement the analysis of population structure with a nonmodel-based approach, a principal coordinate analysis was carried out on the overall microsatellite loci data using GenAlEx software V6.502 (Peakall and Smouse 2006). Principal coordinate analysis enables the identification of the main genetic clusters among the mites sampled; such analysis allows for the identification of drifter mites, i.e., mites found on an atypical host. Once identified, these drifters were subsequently removed from the dataset to prevent bias in the indexes of genetic diversity computed for mites according to their original host.

The number of alleles (*N*_al_), the inbreeding coefficient (*F*_IS_), as well as the observed (*H*_o_) and expected heterozygosity (*H*_e_) were determined within samples for each locus using FSTAT software V2.9.3 (Goudet 2001). We then used GenAIEx V6.502 (Peakall and Smouse 2006) to assess the number of alleles (*N*_al_), the expected heterozygosity (*H*_e_), the adjusted fixation index (*G*_ST_), and Jost’s allelic diversity index (*D*_est_) for each variant of the two honeybee host species for which at least ten samples were available. GenAIEx V6.502 was also used to perform Chi-square tests for Hardy–Weinberg equilibrium (HWE) within samples for each marker and pairwise comparisons of *D*_est_ between variants.

Pairwise distance-based AMOVAs (with 28,000 permutations) were performed on the microsatellite dataset to determine the geographical distribution pattern of the genetic variance of the K1 variants infesting *A. mellifera* and all the haplotypes and variants infesting *A. cerana*. Variants with less than five samples and atypical mites were removed from the dataset to avoid biasing the results. A second AMOVA was then performed to determine the distribution of genetic variability among haplotypes C, J, and K infesting *A. cerana* colonies. The AMOVAs were performed using Arlequin V3.5.1.3 (Excoffier et al. 2005).

Finally, multilocus genotypes were determined with the R tool poppr (Kamvar et al. 2015) in order to compare nuclear genotypes among the mitochondrial haplotypes detected. Comparing the genotypes can provide key information on the origin of the invasive lineage of *V. destructor* from the native populations sampled.

### *Varroa destructor* reproduction

Experimental infestations were performed using standard methods (Dietemann et al. 2013) to determine the ability of *V. destructor* mites to reproduce on both sexes of original and new host species. We used mites from *A.* *cerana* and *A.* *mellifera* colonies collected in the Hangzhou region, without apparent disease symptoms and headed by unrelated queens, to assess *V.* *destructor* reproduction.

Prior to the experimental infestations, mature female mites were collected with paintbrushes after opening drone and worker brood cells of both host species (Dietemann et al. 2013). Mites were collected from eight *A.* *cerana* donor colonies (*N* = 318; Fig. S1a) and from ten *A.* *mellifera* donor colonies (*N* = 383; Fig. S1). All the donor colonies were headed by unrelated queens. Batches of 30 mites of the same host species of origin were kept on 15 caged *A.* *mellifera* adult workers that were previously ascertained to be uninfested. Maintaining the mites on adult workers for two days mimicked the mites’ nonreproductive phase, thereby standardizing their physiological status (Dietemann et al. 2013; Rosenkranz et al. 2010).

The positions of all cells containing worker or drone larvae to be infested experimentally were marked on transparent sheets placed over the combs. These cells were selected based on the developmental stage (L5) of the larvae they contained, which triggers oogenesis in *V.* *destructor* (Dietemann et al. 2013). Once the position of these cells was recorded, the combs were reintroduced into their colonies of origin (*N* = 8 and 4 for *A. cerana* worker and drone brood and *N* = 9 and 4 for *A.* *mellifera* worker and drone brood, respectively). Five hours later, the combs were retrieved, and freshly capped cells were identified using the transparent sheets.

For each comb, freshly capped cells (*N* = 10–15 per colony based on availability of brood at the relevant developmental stage in each colony) were partially opened using a razor blade, and single *V.* *destructor* mites were introduced into each cell using a fine paintbrush. Cell caps were then carefully resealed. In total, 211 worker and 112 drone larvae of *A.* *cerana* and 267 worker and 111 drone larvae of *A.* *mellifera* were infested (Fig. S1).

The experimentally infested brood was reared in an incubator (Yiheng, Shanghai, China) at 34.5 ± 0.5 °C and 70 ± 5% RH (Crailsheim et al. 2013; Williams et al. 2013) to prevent interference by adult host workers (Oddie et al. 2018; Page et al. 2016). The experimental cells were opened one day prior to the expected adult emergence date, i.e., 10, 11, 12, and 13 days after infestation for *A. cerana* worker brood, *A.* *mellifera* worker brood, *A. cerana* drone brood, and *A. mellifera* drone brood, respectively (Chen 2001; Human et al. 2013). Hosts and parasites were retrieved from the cells, and successfully reproducing mites were recorded as those that had produced at least one mated daughter (Dietemann et al. 2013; Lin et al. 2018). A randomly selected subset of the *V. destructor* foundresses used for infestation in the four groups (2 host species × 2 sexes) were collected individually in Eppendorf tubes and stored at − 80 ^°^C until genotyping (see 3.2). This subset represented between 25 and 75% of the samples, corresponding to 215 mites from *A. cerana* colonies and 110 mites from *A. mellifera* colonies (Fig. S1).

### Data analyses

To estimate the number of nondetected variants (all haplotypes confounded), occurring in eastern China, we used asymptotic nonparametric estimators available in the SpadeR package (Chao and Chui 2016). The same analysis was performed to determine whether rare variants of the Hangzhou region subsample used to determine reproductive success were missed. Specifically, we considered the iChao and ACE estimators given the sample size. The cutoff value (*k*) for rare variants was set to 10 for the ACE estimator, and the confidence interval was set to 95%. R version 3.6.1 (R Core Team 2013) was used to run these analyses.

We used generalized linear mixed models (GLMM) to determine whether host species and host sex (worker or drone), as well as host species of origin (from which the mites were collected) and mite variant identity, influenced the reproductive success of the mites. For this purpose, two sets of models were calculated: one using mites from the experimental infestation dataset and the other using only haplotyped individuals (Fig. S1). This allowed the effects of both host species of origin and mite variant identity to be considered. For the variant K1-1/2, we also considered the populations as identified by microsatellite analysis.

Due to a high number of zero values for reproduction (colonies in which foundress mites had no reproductive success), we used zero-inflated models (Zuur et al. 2016). This approach consists of dividing the data into two sets. One set comprises zeroes versus nonzero values (coded as 1 and 0, respectively), such as the proportion of zeroes vs. nonzeros modeled using a Bernoulli approach. The other set comprises the original nonzero values only, which is modeled with a binomial approach. This method has biological relevance, since it allows for distinction between factors that affect the occurrence versus absence of reproductive success and those that affect the proportion of mites that experience reproductive success, where success occurred. According to these models, binomial distribution-based GLMM were used to analyze the proportional data (hosts showing reproductive success) within the complete sample.

Host species (*A. mellifera* and *A. cerana*), host sex (worker and drone), and host species of origin (mites sampled from *A. mellifera* or *A. cerana* colonies) were considered fixed factors. The identities of the colonies used for the experiments were considered random factors. However, this factor was omitted from the models when its variance component approached zero, indicating limited influence of this parameter. In the models, variance was partitioned between the factors according to the type III marginal approach, in which each factor is adjusted for every other factor.

Models were calculated with all combinations of fixed and random factors and all possible interactions between fixed factors. Model selection was based on the value of coefficient errors and then on Akaike Information Criteria (AIC). The models yielding coefficient errors with a degree of magnitude higher than the coefficients themselves were considered unreliable and discarded. Of those remaining, the models with the lower AIC were selected. For the binomial part of the zero-inflated models, fit was also assessed based on QQ plots before considering the AIC. The statistical significance of differences between host species, host sex, and mite origin, as well as their interactions, was obtained from the estimated means (lsmeans function).

In order to obtain a population-wide estimate, reproductive success was assessed based on all cases of experimental infestation, including those in which the infested brood experienced developmental disturbance or died. Given that the conclusion drawn from multiple inferences was unlikely to be erroneous when at least one of the inferences was, we corrected for false discovery rate (Benjamini and Hochberg 1995) when significant differences occurred. This procedure estimates the expected proportion of errors among the rejected hypotheses. R (version 3.4.2; R Core Team 2013) was used for these analyses (car, multcomp, lsmeans, and glmmadmb packages).

## Results

### Geographic distribution, genetic diversity and phylogeny of *Varroa destructor* based on mtDNA

Based on the concatenated mtDNA sequences of the *cox1* and *cytb* genes, 17 variants of five haplogroups and three haplotypes were identified in the 463 mites collected from *A.* *cerana* colonies. The estimated number of variants in the region ranged from 24 to 28, with a 95% confidence interval of (18, 51) and (19, 68), respectively, for the two richness estimates computed (see Table S3). Mites (*N* = 362) collected from *A.* *cerana* colonies in central and eastern China clustered into the Korea haplotype (Figs. 1, 2). Their sequences were highly similar, with only one to seven substitutions in the 1806-bp sequence (Table S4). Based on these substitutions, the mites segregated into 14 variants of the K haplotype, including two known variants (K1-1/2, K1-3; Navajas et al. 2010) and 12 reported here for the first time, namely K1-5, K1-6, K1-7, K1-8, K1-9, K1-10, K1-11, K1-12, K1-13, K1-14, K1-15, and K2-1. The K2-1 mite was sampled from an *A. cerana* colony in Ya’an, at the western border of the region surveyed, and despite clustering within the Korean haplotype, it was distinct from all other known K haplotypes (Figs. 1, 2, Table S4). This mite was thus designated as variant 1 of a new haplogroup (K2).

K1-5, K1-6, and K1-12 clustered with K1-1/2 due to the high sequence similarity (reaching 99.95%), the differences between variants being due to single substitutions (Fig. 2, Table S4). Of all the *V.* *destructor* K1 variants, K1-5 was the most prevalent, accounting for 52.4% of the mites sampled (189 out of 361) from *A. cerana* colonies. Seventy-three mites (21.1%) from *A.* *cerana* colonies in Hangzhou, Chun’an, Fuyang, Lin’an, Yuanpu, Shengzhou, Jinhua, Wencheng, and Taishun of Zhejiang Province, southeast China, and one mite collected in Tai’an of Shandong Province, eastern China (Fig. 1a), were identified as the K1-1/2 variant and clustered with the mite found in *A. mellifera* colonies (Fig. 2). The K1-4 variant reported by Navajas et al. (2010) in Yunnan Province, southwest China, was not found in our samples.

The unrooted phylogenetic tree, including one representative sample of each identified haplotype (Fig. 2), showed two main clusters. One comprised the C2, C3, and C4 haplogroups; the other comprised haplotypes J, K, and V together with mites from the C1 haplogroup. C1 mites clustered with mites from the V1 haplogroup. J and K haplotypes formed the second large cluster. The K cluster was divided into K1 and K2 subclusters. Within the K1 cluster, geographic regions could be recognized with K1-8, K1-9, and K1-11 occupying the more southern regions and K1-3 occupying the more northern region of the K1 distribution area (Fig. 1a). K1-1/2 and K1-5 were spatially more widespread but were not found in the southern region of the K1 area (Fig. 1a).

**Fig. 3:**
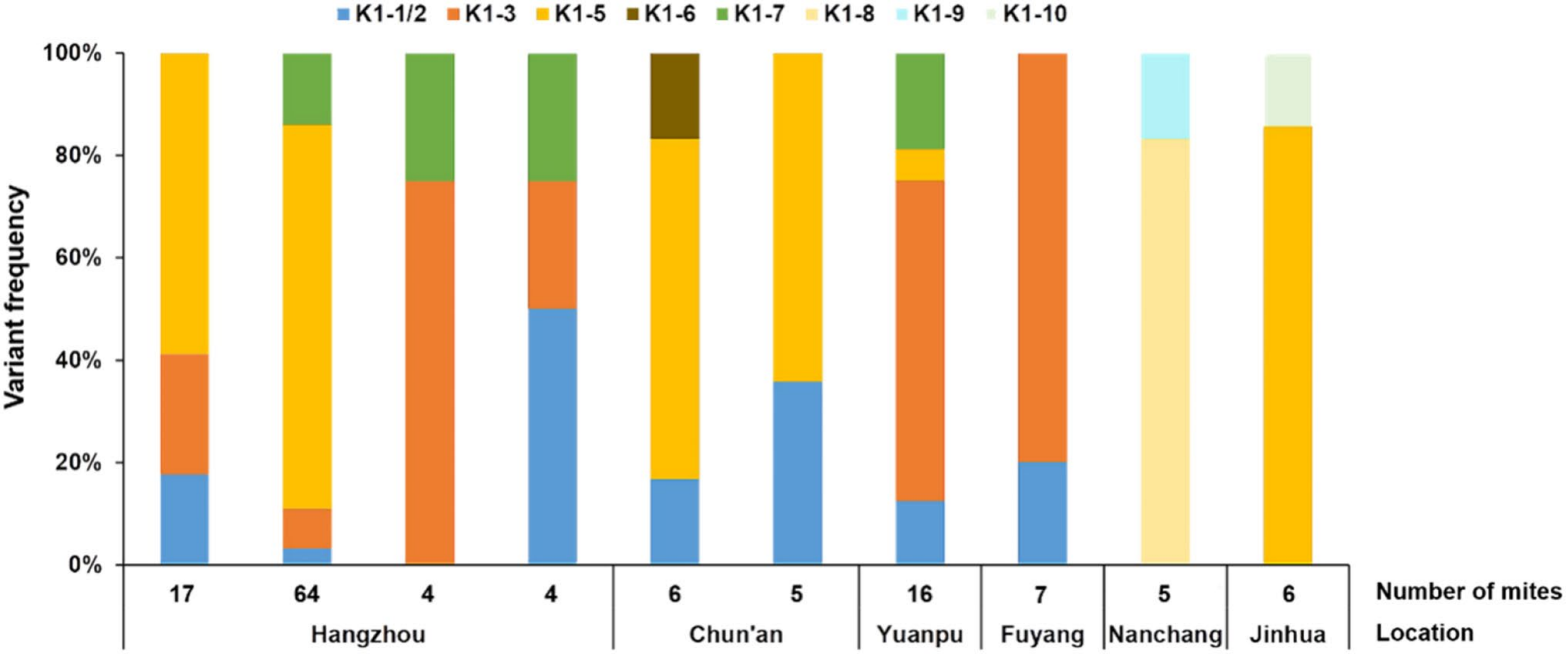
Variant frequencies of *Varroa destructor* K1 haplogroup in ten *Apis cerana* colonies showing infestation with multiple variants. Each bar represents the mite variants found within a colony. The location of colonies and the number of mites collected are indicated

In six of the 20 sampled localities, infestations of *A. cerana* colonies with multiple variants were detected. Up to four K1 variants of *V. destructor* co-occurred in a single colony (Fig. 3). The percentages of colonies in which singly infested cells were detected or in which cells were infested by two, three, and four variants were 36.3, 51.0, 8.8, and 3.9, respectively.

Nineteen mites collected from Guangzhou, six from Zhanjiang, and one from Maoming, southern China, were determined to be of the China haplotype (Figs. 1, 2). One of these mites, collected from Guiyang, southwest China, did not belong to a known haplogroup. This mite clustered on a distinct branch of the phylogenetic tree with high bootstrap value and was therefore designated as variant 1 of a new haplogroup (C4; Fig. 2, Table S4).

Seventy-four mites collected in Antu, Jilin Province, Northeast China, clustered with the Japan/Thailand haplotype (Figs. 1, 2) and had only one nucleotide difference to the known J1-3 variant (Table S4). Since these mites occupied a distinct branch of the tree with a high bootstrap value (Fig. 2), we classified them as belonging to variant 1 of a new haplogroup (J2).

Except for two individuals, all mites collected from *A.* *mellifera* colonies in China belonged to the K1-1/2 variant of *V. destructor*. The two exceptions were collected in Hangzhou, belonged to the K1-5 variant, and were genetically identical to the mites of this same variant collected from *A. cerana* colonies (Fig. 2, Table S4).

### Genetic diversity, population structure, and gene flow in *Varroa destructor* based on DNA microsatellite markers

Considering all mites genotyped, locus VjL3B2 was monomorphic (189/189 in all samples), whereas the five others were polymorphic (Tables S5, S6). Vj 275 was highly variable and difficult to interpret (Tables S5, S6; Solignac et al. 2005). These two loci were thus excluded from further analyses. After their exclusion, the number of alleles of selected loci ranged from 9 to 16 (Table S5).

Selected loci showed high inbreeding coefficients (*F*_IS_ > 0.8) and deviated significantly from HWE (Table S5). Allelic richness, *H*_o_ and *H*_e_ ranged between 12.9 and 27.6, 0.09 and 0.5, and 0.6 and 0.9, respectively (Table S5). *G*_ST_ values were low to moderate, suggesting a degree of fixation in some populations. Inspection of allele frequencies showed that two of the four alleles were fixed in the K1-1/2 variant infesting *A. mellifera* (Table S6). *D*_est_ values were moderate to high. Significant deviations from random mating were observed among loci within populations and among populations within loci (Tables 3, S5). Microchecker did not detect allele dropout, and INEST detected a minimal frequency of null alleles (< 0.003).

**Fig. 4:**
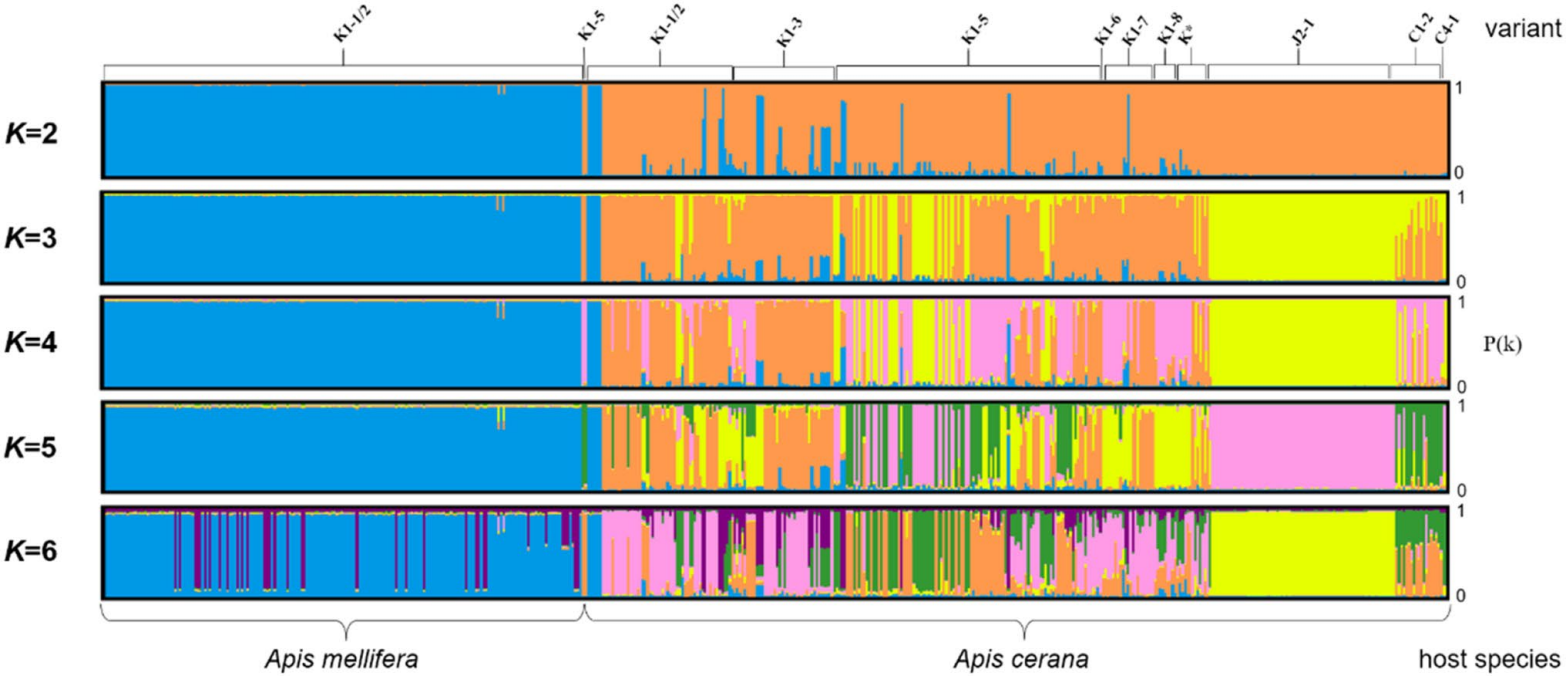
Results of population structure InStruct analysis of *Varroa destructor* mites from *Apis mellifera* and *Apis cerana* for various genetic clusters between *K *= 2 and *K *= 6. Each cluster is represented by a different color. Each bar represents an individual mite (*N* = 531) and is arranged according to variant identity (top) and host species (bottom). The *Y*-axis of each horizontal bar represents the likelihood *P*(*k*) that each individual belongs to a genetic cluster. K* includes individuals from variants K1-9 to K1-15 and K2-1

InStruct analysis assigned individuals to genetic clusters (*K*) without prior spatial information. The Gelman–Rubin statistic for all values of *K* was < 1.10, indicating a good convergence in both log-likelihood and selfing rates. The most likely number of genetic clusters represented in the dataset in the range *K *= 1 to *K *= 25 was *K *= 2 (Δ*K *= 176). For *K *= 2, genetic clusters corresponded to host species, with the exception of six mites belonging to the cluster typical for *A. mellifera* K1-1/2 mites found in *A. cerana* and two K1-5 mites belonging to the *A. cerana* mite cluster found in two *A. mellifera* colonies (Fig. 4). These mismatches occurred for all other values of *K*. Excluding these individuals, mites of the K1 haplogroup found in *A.* *cerana* colonies were generally more genetically admixed than those collected from *A.* *mellifera* (Fig. 4). For *K *= 3, J2-1 and some individuals from several K variants formed a new cluster with introgression in several other *A. cerana* mite variants and haplotypes. For *K *= 6, a J2-1 specific cluster appeared with minimal introgression in other haplotypes. *A.* *mellifera* K1-1/2 mites remained a mostly homogeneous cluster with minimal introgression until *K *= 5 and showed higher diversity for larger values of *K* (Fig. 4). Examining *K* values higher than six introduced genetic heterogeneity with no clear biologically relevant grouping which are therefore not shown.

**Fig. 5:**
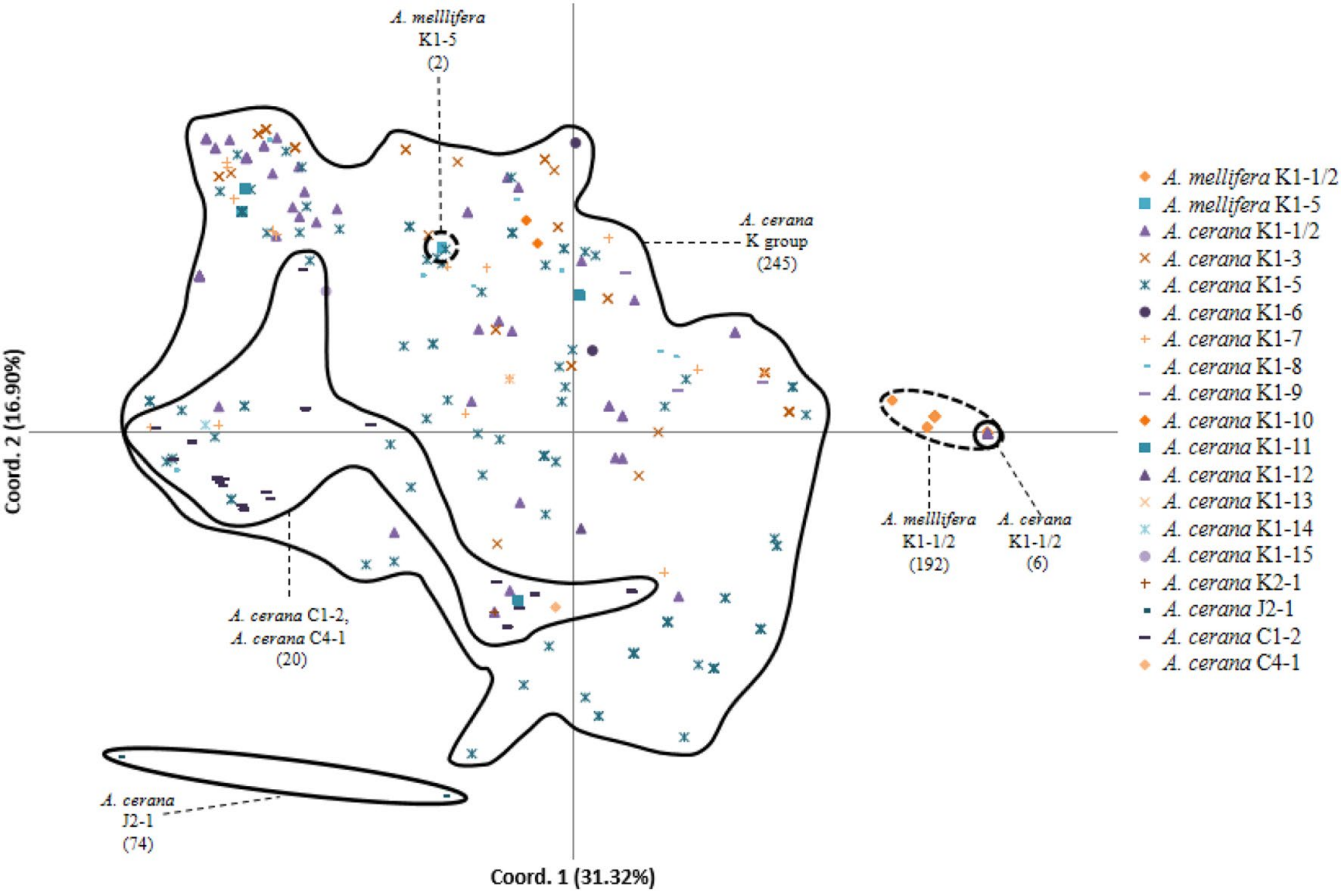
Principal coordinate analysis of *Varroa destructor* mites infesting *Apis mellifera* and *Apis* *cerana.* Hand-drawn shapes delineate the minimal space occupied by each mite haplotype collected from each host species. Shapes with dashed lines indicate mites collected from *A. mellifera* colonies; those with full lines indicate mites collected from *A. cerana* colonies. Variants K1-1/2 to K1-15 and K2 collected from *A. cerana* did not segregate and were thus grouped within one shape for clarity. Sample size is given between parentheses for each group of individuals delineated

The mismatched host–parasite associations (individuals belonging to the *A.* *cerana* mite genetic cluster collected from *A. mellifera* colonies and individuals belonging to the *A.* *mellifera* mite genetic cluster collected from *A. cerana* colonies) identified by the *K* analysis were also observed in the output of the principal coordinate analysis. Except for six individuals, the *A. cerana* K1-1/2 mites segregated away from the *A. mellifera* K1-1/2 cluster in the space delineated by principal coordinates 1 and 2 (Fig. 5). Conversely, mites belonging to the *A.* *cerana* K cluster included two individuals collected in *A. mellifera* colonies. Apart from these exceptions, the position of most *A. cerana* mite variants belonging to the K haplotype overlapped in the space delimited by principal coordinates 1 and 2. This large cluster also included mites of the C haplotype. In contrast, the *A. cerana* mites of the J haplotype clustered away from the other *A. cerana* and *A. mellifera* mites.

For *V. destructor* variants infesting *A. cerana* and *A. mellifera*, the AMOVAs indicated that the largest genetic variance (40–70%) was at the sampling location level. A further quarter of the variation was found among regions in *A. cerana* mites, whereas this level showed no structuring in *A. mellifera* mites. The lowest variance in *A. cerana* mites was among sampling locations within regions (Table 1). Considering *A. cerana* mites only, half of the genetic variance was within haplotypes, whereas a third of the variance was found among them (Table 2).

**Table 1:** Results of two pairwise distance-based AMOVAs on microsatellite data

Host species	Source of variation	d.f.	Sum of squares	Variance components	Percentage of variation	Significance^†^
*Apis cerana*	Among regions	2	190.92	0.48	24.49	**
	Among locations within regions	16	149.44	0.30	15.22	***
	Among individuals within locations	320	625.05	0.78	40.18	***
	Within individuals	339	132.50	0.39	20.1	***
*Apis mellifera*	Among regions	2	1.97	− 0.01	− 4.63	n.s.
	Among locations within regions	9	9.68	0.04	25.23	***
	Among individuals within locations	180	43.97	0.11	69.85	***
	Within individuals	192	3.00	0.02	9.55	***

Data show the level of genetic structuring of *Varroa destructor* parasitizing each host species at various spatial levels (regions: northern, central and southern parts of eastern China; locations: see Fig. 1)

^†^n.s.: nonsignificant; ** *P *< 0.01; *** *P *< 0.001

**Table 2:** Results of a pairwise distance-based AMOVAs on microsatellite data

Source of variation	d.f.	Sum of squares	Variance components	Percentage of variation	Significance
Among haplotypes	2	188.685	0.6522	32.91	***
Among individuals within haplotypes	320	730.689	0.95362	48.11	***
Within individuals	323	121.5	0.37616	18.98	***

Data show the level of genetic structuring of *Varroa destructor* parasitizing *Apis cerana* among and within haplotypes

One hundred and eighty-three microsatellite multilocus genotypes were identified in the sample (Table S7). Despite high sample numbers, the K1-1/2 mite population infesting *A.* *mellifera* (*N* = 192) and the J2-1 population infesting *A. cerana* (*N* = 73) were composed of only four and two genotypes, respectively. In contrast, the other haplotypes with similar number of mite samples (range: 53–106) were composed of 37–66 genotypes (Table S7). Genotypes of K1-1/2 mites infesting *A. mellifera* were different from those of K1-1/2 mites infesting *A.* *cerana*. Even though the alleles in these populations were the same, with those of *A. mellifera* being a subset of those found in *A. cerana*, their combinations were different and hence generated the different genotypes observed (Table S6).

For the assessment of the diversity indexes, the atypical mite–host associations identified by the *K* and principal coordinate analyses were removed from the dataset of their respective host species. None of the variants were at HWE (*F*_IS_ > 0; Table 3). The overall heterozygosity in *V. destructor* mites infesting *A.* *cerana* colonies was higher compared to those infesting *A.* *mellifera* colonies, except for J2-1, which indicated very low heterozygosity (Table 3). Correspondingly, J2-1 mites infesting *A. cerana* colonies had the highest *F*_IS_, followed by K1-1/2 mites infesting *A. mellifera*. K1-5 and K1-1/2, the most frequent variants found in *A. cerana* colonies, had the highest mean number of alleles. The number of alleles and expected heterozygosity of K1-1/2 mites from *A.* *mellifera* colonies were generally lower than those of the K1-1/2 mites collected from *A.* *cerana* colonies (Table 2).

**Table 3:** Genetic population structure summary metrics and statistics based on microsatellites of *Varroa destructor* mites belonging to several haplotypes and variants collected from Chinese *Apis* *mellifera* and *Apis cerana*

Host species	Mite variant	*N*_a_^†^	*N*_c_	*N*_i_	*N*_al_	*H*_e_	*F*_IS_
*A. mellifera*	K1-1/2	12	19	192	1.40 ± 0.25	0.06 ± 0.06	0.95 ± 0.04
Overall				192	1.40 ± 0.25	0.06 ± 0.06	0.95 ± 0.04
*A. cerana*	K1-1/2	6	9	53	7.40 ± 1.97	0.62 ± 0.16	0.73 ± 0.01
	K1-3	3	4	40	5.40 ± 1.21	0.58 ± 0.15	0.74 ± 0.05
	K1-5	6	9	106	7.40 ± 1.81	0.62 ± 0.16	0.68 ± 0.04
	K1-6	1	2	2	–^‡^	–	–
	K1-7	2	4	21	5.60 ± 1.40	0.59 ± 0.15	0.65 ± 0.05
	K1-8	1	2	10	2.80 ± 0.58	0.45 ± 0.11	0.38 ± 0.18
	K1-9 to K1-15	1	1	1-3	–	–	–
	K2-1	1	1	1	–	–	–
	J2-1	1	4	74	1.20 ± 0.20	0.10 ± 0.10	1.00 ± 0.10
	C1-2	2	2	19	4.20 ± 1.07	0.49 ± 0.17	0.50 ± 0.14
	C4-1	1	1	1	–	–	–
Overall				339	10.60 ± 2.69	0.65 ± 0.16	0.76 ± 0.02

^†^*N*_a_: number of apiaries; *N*_c_: number of colonies; *N*_i_: number of individual mites; *N*_al_: mean number (± se) of different alleles over the six microsatellite loci; *H*_e_: mean expected heterozygosity ± se; *F*_IS_: inbreeding coefficient

^‡^ ‘–’ indicates cases in which too few individuals (< 10) were found to calculate the genetic diversity indexes

Pairwise comparison of *D*_est_  among *V. destructor* variants infesting *A. cerana* and *A.* *mellifera* colonies revealed significant differences after correction for multiple comparisons (Table 4). The *D*_est_ values among *V.* *destructor* K variants infesting *A.* *cerana* were moderate to high, ranging between 0.18 and 0.63. The estimates among *V.* *destructor* K and C variants infesting *A. cerana* and the variant infesting *A. mellifera* were higher overall, ranging between 0.60 and 0.89. J2-1 showed the highest pairwise *D*_est_ values when combined with most variants. The paired estimate for the comparison between J2-1 and K1-8 was the highest overall (0.91).

**Table 4:**
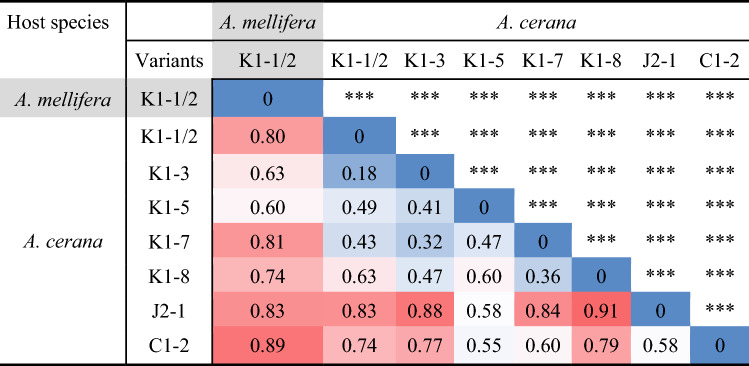
Pairwise *D*_est_ analysis among *V. destructor* variants infesting *A.* *mellifera* and *A.* *cerana* colonies. Cell background color from blue to red correlates with increasing *D*_est_ estimate values. *** *P* < 0.001

### *Varroa destructor* reproduction

**Fig. 6:**
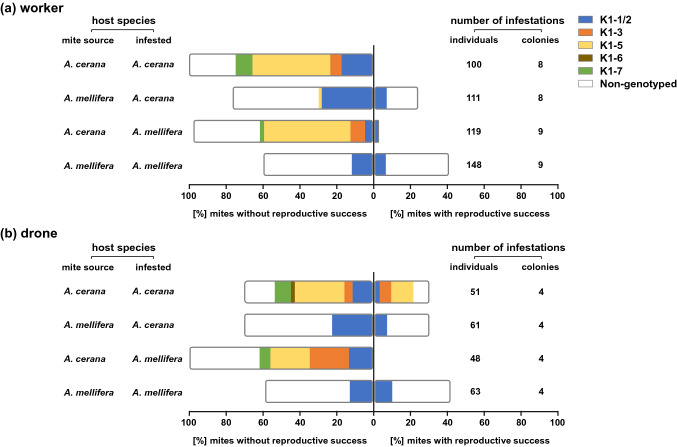
Percentage of *Varroa destructor* foundress mites without and with reproductive success after experimental infestation of **a** worker and **b** drone brood of *Apis cerana* and *Apis* *mellifera.* The original host species of mites collected for experimental infestation and the host species experimentally infested are indicated to the left of the bars. Number of individuals and colonies infested are indicated on the right

Mites collected from *A. cerana* in the Hangzhou region and used experimentally to infest worker and drone brood cells of both host species belonged to variants K1-1/2, K1-3, K1-5, K1-6, and K1-7. The estimated number of variants in this region was seven, with a 95% confidence interval of (6.1, 19.6) for the two richness estimators computed (for details, see Table S8). The majority of foundresses were fertile (81–100%, mean ± s.d = 92.1 ± 7.8%) when placed on *A. cerana* drone brood, but only a third of them achieved reproductive success (i.e., produced at least one mated daughter) (Fig. 6). Only one out of 100 and two out of 119 reproduced on other brood types, i.e., on worker brood of *A.* *cerana* and *A.* *mellifera*, respectively. One of these three mites produced a viable daughter (Fig. 6a). The haplotype of these three individuals was identified as K1-1/2, and microsatellite analysis showed that these mites belonged to the invasive mite population infesting *A. mellifera* (Figs. 4, 5).

The majority of mites (58–84%, mean ± s.d = 68.6 ± 12.2%) collected in *A. mellifera* colonies were fertile when placed on brood of both host species and both sexes, with between 25 and 40% achieving reproductive success (Fig. 6). Two mites collected from *A.* *mellifera* were identified as K1-5 variants (Figs. 4, 5). These mites did not reproduce (Fig. 6a). The five mites found in an atypical host were excluded from the dataset for modeling reproductive success.

Absence versus occurrence of reproductive success was best modeled when including the factors of the original host species and host species into which the mites were experimentally introduced. Both factors affected reproductive failure significantly (Table S9). When the interaction between these factors was considered, the reproductive success of *A. cerana* mites was significantly less frequent when infesting both hosts compared to *A. mellifera* mites (Table S9). The probability of successful reproduction of *A. mellifera* mites was lower on *A.* *mellifera* than on the original host, *A. cerana*. In *A. cerana* mites, success was less likely on *A. mellifera* hosts than on *A. cerana* hosts. When considering only cases in which reproductive success was achieved (by mites originating from *A. mellifera* in all categories of brood and by *A. cerana* mites in *A.* *cerana* drone brood), the proportion of foundresses achieving success was best modeled by the factor of host species infested. A significantly greater proportion of mites achieved success in *A. mellifera* hosts compared to *A. cerana* hosts.

When mite variant and microsatellite population (which distinguishes K1-1/2 infesting *A.* *mellifera* from K1-1/2 infesting *A. cerana*) were considered as factors of mite identification rather than host species of origin, host sex alone best modeled the absence versus presence of reproductive success. Mites infesting worker hosts had a significantly lower probability of reproductive success than those infesting drones, irrespective of host species infested and variant identity. When only cases of reproductive success were considered, the same factor best modeled the proportion of foundresses achieving success. This proportion was higher in drone hosts, but the difference between sexes was not significant (Table S9).

## Discussion

Our large-scale screening of *V. destructor* mites in managed populations of *A. cerana* in eastern China led to the identification of distribution ranges of C, K, and J haplotypes from the south to the north of eastern China. In each haplotype, new haplogroups and variants were identified, with likely more remaining to be detected in future surveys. The *V. destructor* K1-1/2 haplotype that has shifted host to *A. mellifera* was found on its original host, *A. cerana*. However, microsatellite analyses identified differences between mites infesting original and new hosts. These mite populations showed unidirectional gene exchange, and their reproductive abilities differed. The lineage infesting *A. mellifera* was able to reproduce on all host sexes and species tested, whereas all variants infesting *A.* *cerana* were only able to reproduce on drone brood of their original host.

### Distribution, genetic diversity, and taxonomy of *Varroadestructor* in eastern China

MtDNA genotyping revealed three *V. destructor* haplotypes infesting *A.* *cerana* in eastern China: C, K, and J. These haplotypes occurred in the southern, central, and northern regions of eastern China, respectively, with no apparent overlap. Our results thus confirm the description of the spatial distribution of C and K haplotypes reported in previous studies (Anderson and Trueman 2000; Navajas et al. 2010), completing it through to the wide geographic area sampled (Fig. 1). For instance, we report for the first time the occurrence of mites from the Japan haplotype in mainland China. The occurrence of the J haplotype in the northernmost region sampled—close to the region in which it is currently assumed that the host shift of the invasive lineage of K1-1/2 from *A.* *cerana* to *A. mellifera* took place (in southeastern Russia; Rosenkranz et al. 2010)—was unexpected and suggests that the distribution of the K haplotype is not continuous. Alternatively, the distribution range of the J haplotype may be embedded or neighboring that of the K haplotype, which could be resolved by a higher sampling effort in the region.

The phylogenetic tree showed with relatively high support that the mites of the J haplotype sampled did not cluster with known J haplogroups; we therefore designated them as variant 1 of a new J2 haplogroup (J2-1). J2-1 mites displayed poor allelic diversity combined with a high degree of homozygosity. Since the range of values found is similar to those for the invasive K1-1/2 lineage, it is likely that this population also experienced through a genetic bottleneck. Such a bottleneck could have occurred after an introduction of infested *A. mellifera* from Japan (Chen 2001), with subsequent changes due to genetic drift or to the adaptation of the local *A*. *cerana* host. However, this is unlikely since the mite haplotype found in *A. mellifera* colonies in Siping and Baishan, Jilin Province, is K1 (this study, Fig. 1; Zhou et al. 2004). The introduction hypothesis would only hold true if the J haplotype that may have originally infested *A. mellifera* was later outcompeted and superseded by the invasive K1-1 lineage, as has been reported in other parts of the world (Garrido et al. 2003; Ogihara et al. 2020). The most parsimonious explanation is that the J2-1 haplotype is the original parasite of this *A. cerana* population. This hypothesis is supported by the lack of any other mite haplotype, haplogroup, or variant in our survey of this northern region of eastern China. At this latitude, representing the edge of natural *Varroa* spp. and *A. cerana* distribution (Navajas et al. 2010; Pesenko et al. 1990; Radloff et al. 2010), the time window of drone brood production is only a few weeks, potentially leading to very low colony infestation rates. These extreme conditions are probably at the limit of what *V.* *destructor* can tolerate and could have led to the selection of a particularly well-adapted variant (J2-1). Declining genetic diversity on the core–edge gradient of distribution areas due to smaller effective population size and greater geographical isolation has been shown in numerous other organisms (Eckert et al. 2008).

Furthermore, new haplogroups were identified for haplotypes C and K based on their distant position from known haplotypes in the phylogenetic tree. The two mites found in the central and southeastern regions of China were named variants 1 of haplogroups C4 and K2 (C4-1, K2-1), respectively.

The K1-4 variant found in *A. mellifera* by Navajas et al. (2010) was not found during our survey, neither on *A.* *cerana* nor on *A. mellifera*. K1-4 mites most likely belong to a population restricted to southwestern China that we have not sampled. In contrast, we found 17 variants of the K1 haplogroup, of which 12 are newly described, but with more yet to be discovered based on estimators of species richness (Table S3). The high genetic diversity within the K haplotype indicates a high evolutionary potential (McDonald and Linde 2002), with an increased probability that one variant becomes able to use alternative host types (Vignuzzi et al. 2006). Among the known variants, K1-1/2 (Anderson and Trueman 2000; Navajas et al. 2010) was among the most prevalent and is here reported infesting *A. cerana* for the first time outside Korea. Phylogenetic analysis showed that the endemic K1 variants infesting *A. cerana* and the host-shifted K1-1/2 lineage belonged to the same cluster, indicating a recent common ancestor. The low genetic diversity of the invasive lineage measured in eastern China is consistent with genetic bottlenecks due to host shift (Solignac et al. 2005). Indeed, the fixation of alleles at several loci was not found in mite populations infesting the original host, including the K1-1/2 mitochondrial variant (Tables 3 and S6). Nuclear DNA-based genotype analyses (Table S7, Figs. 4 and 5) indicated that the K1-1/2 variants infesting each host are genetically distinct. This difference in genotypes could result from a divergence and adaptation of the invasive lineage to their new host following the host shift and/or from a different origin of the invasive population. A more intensive genotyping effort in the region in which the host shift is supposed to have taken place (Traynor et al. 2020) is required to unravel these possibilities.

### Reproduction systems

The richness estimators indicated that the reproductive success of at least two rare variants was not determined (Table S8). Among those for which data were obtained, the differences in genotypes between the populations of the K1-1/2 variant infesting each host species were associated with clear phenotypic differences. The K1-1/2 variant hosted by *A.* *cerana* was only able to reproduce on drone brood of this species, whereas that hosted by *A.* *mellifera* could reproduce on brood of all sexes and species. Modeling indicated that the reproductive success or failure of foundress mites was determined by the factors of host species infested and mite origin, highlighting the peculiar reproductive ability of the invasive mite lineage. The inability of *A.* *cerana* mites to reproduce on *A. mellifera* brood led to the achievement of significantly lower reproductive success on *A.* *mellifera* brood than on the brood of its original host, confirming their high host specificity (Anderson 1994; Li et al. 2019; Rath 1999). In contrast, the probability of *A. mellifera* mites reproducing successfully on brood of the new and original hosts was not significantly different (Table S9), suggesting that the host-shifted lineage is well adapted to both host species.

When considering only colonies in which mites achieved reproductive success (using the binomial model; Table S9), the factor determining the proportion of reproductively successful foundresses was the host species infested. A higher proportion of mites reproduced on *A.* *mellifera* brood. Since no *A. cerana* mites reproduced on the brood of *A. mellifera*, this points to the high ability of the host-shifted lineage to exploit its new host and to the low reproductive success of *A. cerana* mites in their original host. This is in line with reproductive abilities observed in natural infestations (Wang et al. 2020) and might be due to as yet unknown resistance mechanisms.

When more precise genotype information was used instead of host species of origin to indicate mite lineage identity, only host sex was seen to affect the occurrence of reproductive success significantly, thus failing to flag the peculiar reproductive abilities of the invasive lineage. This may be due to a dilution effect given the increased number of categories considered (six variants vs. two host species), combined with a reduction in sample size (not all individuals used in infestations were genotyped). Overall, a significantly greater proportion of mites achieved success in *A. mellifera* hosts than in *A. cerana* hosts and drone brood was confirmed to promote mite reproduction more than worker brood, irrespective of host species. The lack of reproduction of *A. cerana* mites on worker brood is probably due to this trait being maladaptive in *A. cerana* (Page et al. 2016).

### Population structure, population dynamics, mite dispersal, and gene flow

#### *Apis cerana* vs. *Apismellifera* mites

The mite haplotypes and variants infesting *A. cerana* revealed greater genetic diversity and less inbreeding than those collected from *A. mellifera* (Table 3), which is in line with *Varroa* spp. populations infesting *A. cerana* in other countries (Vietnam, Philippines: Beaurepaire et al. 2015; Papua New Guinea: Roberts et al. 2015; Thailand: Dietemann et al. 2019). Investigation of spatial genetic structuring showed that for mites infesting both host species, the level with the most variance was within sampling locations (Table 1). For *A. cerana* mites, this pattern is likely due to the high number of variants co-occurring at each locality; this was supported by the AMOVA based on the taxonomic units, which showed higher variance within compared to among haplotypes (Table 2).

In these mites, the genetic variance between regions was higher than among sampling locations within these regions, which reflects the distribution of the haplotypes C, K, and J. For *A.* *mellifera* mites, the high variance at the location level suggests a local co-occurrence of several lineages. Indeed, we found four genotypes in the samples collected (Tables S6 and S7), in line with the recent discovery of fine-scale genetic variation in the invasive lineage (Beaurepaire et al. 2017; Dynes et al. 2016). The absence of structure at the regional level indicates less differentiation over larger spatial scales, which could be due to the frequent translocations of this host within the country (Zheng et al. 2018), thereby spreading the various genotypes homogeneously. By contrast, *A. cerana* colonies are not displaced and are only trapped locally (Zheng et al. 2018). Therefore, the identified population structure of *A. cerana* mites likely reflects the natural biogeography and dispersal patterns of *V. destructor*.

#### J haplotype

The analysis of the nuclear DNA of J2-1 mites showed that gene flow between this variant and the other genetic groups is low, as indicated by high *D*_est_ pairwise comparison values (Table 4) and by the principal coordinate analysis, in which the principal coordinate 2 somewhat segregated this cluster (Fig. 5). In contrast, the J2-1 mites were included, together with all other *A.* *cerana* mites, within one of the two most likely genetic clusters by the InStruct analysis. Separation of this haplotype into a genetic cluster was obtained when three or more clusters were hypothesized, although this was not highly specific as it included introgressed individuals from several K variants. The J2-1 variant segregated completely when six genetic clusters were considered (Fig. 4). It is thus not clear how much gene flow occurs between the J haplotype and the C or K haplotypes. Increased sampling density in the region separating the locations screened here could clarify this issue.

#### C haplotype

In the mtDNA phylogeny, C4-1 clustered with C2 and C3 haplogroups and was well separated from the C1/V1 cluster. This phenomenon has already been observed in Navajas et al. (2010) and could be due to the isolation of these populations by the Hengduan Mountains bordering Vietnam and Thailand. Since these new variants are only represented by a single mite each, wider surveys are need to determine their distribution range and prevalence. Although the genetic distance of C4-1 to the K1 cluster is higher than that between K1 and J2-1 (Figs. 2, 4, Table 4), the C and K haplotypes overlapped in the principal coordinate analysis, and InStruct analysis showed high degrees of introgression for all values of *K* (Fig. 4). This pattern was further highlighted by comparison of pairwise differentiation indexes, which were higher for pairs including J haplotypes than those including C haplotypes (Table 4). This could be due to a higher degree of gene flow between the C and K haplotypes than between the K and J haplotypes, possibly due to dispersal over a more permeable geographic barrier in the southern region of eastern China.

#### K haplotype

As a result of sympatry, several (up to four) K1 variants were occasionally found in the same colony. This intrapopulation mix (Huyse et al. 2005) could lead to interbreeding and gene flow between variants. Given the absence of private alleles in genetically similar variants (Table S6), it is not possible to identify hybrids, as was recently done in Thailand where *V. destructor* and *Varroa jacobsonii* co-occur (Dietemann et al. 2019). Principal coordinate and InStruct analyses suggest that K1 variants in *A. cerana* belong to a single genetic cluster, which supports the idea of a lack of reproductive isolation between these variants. Pairwise *D*_est_ values among K1 variants infesting *A. cerana* were low and confirmed a moderate differentiation (Tables 2, 4). Overall, this suggests that gene flow occurs within this haplogroup. Males could breed with daughters of foundress mites from different mitochondrial variants that invaded the same cells. Brood cells hosting several foundresses accounted for 16% of infestations by the K haplotype (Wang et al. 2020), indicating that this is not a rare phenomenon. Such a high frequency could be due to the short period during which drones are produced in *A. cerana* and to the relatively small number of drones reared (Koeniger et al. 2011).

Microsatellite analyses showed that, with comparable sampling effort, introgression between host-shifted and nonshifted mite populations took place almost exclusively in mites collected from *A. cerana* colonies (Fig. 4). This unidirectional gene flow is likely to derive from a partial barrier caused by the differential reproductive patterns between the mite populations observed here. *A. cerana* mites, including the K1-1/2 variants, were unable to achieve reproductive success on *A. mellifera* brood when drifting or when introduced in cells of the alternate host. By contrast, the host-shifted lineage is able to reproduce on the original host when the brood survives infestation (Dietemann et al. 2019; Li et al. 2019; Lin et al. 2018), thereby enabling its offspring to breed with that of mites from nonshifted populations. This hybrid offspring evidently does not acquire the ability to reproduce on worker brood of the original host, given that our experimental infestation of hybrids in *A. mellifera* never resulted in reproductive success. However, this could be the case if the genes coding for the traits that decrease host specificity become introgressed. Were such hybrids able to reproduce successfully on worker brood, damage to *A. cerana* colonies would be expected.

Indeed, this trait of the host-shifted lineage has been hypothesized as the main detrimental factor to *A. mellifera* colony health (Evans 2019; Lin et al. 2018; Rosenkranz et al. 2010). The apparent absence of gene flow from the endemic to the host-shifted population also has the positive consequence that the latter remains of limited genetic diversity and thus of low evolutionary potential (McDonald and Linde 2002). This unidirectional gene flow also sets the stage for speciation of the host-shifted lineage once enough differentiation has occurred (Gray and McKinnon 2007; Smadja and Butlin 2011). The microevolutionary processes identified in this study thus link with macroevolution in this particular parasite (Huyse et al. 2005).

Whether the population structure detected reflects isolation by distance or obstacles to dispersal and gene flow remains to be determined. In the framework of this study, we were unable to determine whether the phylogeny of *V. destructor* matched that of their host because of local coevolution. Local coevolution, however, seems unlikely due to the lack of geographical patterns in *A.* *cerana* haplotype distribution in China (Gong et al. 2018; Ji et al. 2011). This is in line with the mismatch observed in Thailand, where the population structure of host and parasites has been attributed to biogeographic history and recent dispersal rather than to local coevolution (Rueppell et al. 2011).

### Drifting mites and host shifts

The K1-4 variant (not found in our screening) has previously been found in *A. mellifera*, but it was not established whether its presence resulted from drifting (Dietemann et al. 2019; this study) or if it reproduced in this host (Navajas et al. 2010). Here, the exclusive presence of the host-shifted K1-1/2 lineage in *A*. *mellifera* colonies and its low genetic diversity in the region screened indicate that no other local *A*. *cerana* K1 lineage has shifted host. This strengthens the hypothesis of a barrier to gene flow from the nonshifted variants to the host-shifted lineage.

On some occasions, mites typical of *A. mellifera* were found in *A. cerana*, and inversely, mites typical for *A. cerana* were found in *A. mellifera*. Two K1-5 mites collected from *A.* *mellifera* colonies clustered with mites from this variant collected from *A. cerana*, which is likely to have resulted from mites drifting between colonies of both species. This variant was not able to reproduce when introduced in *A.* *mellifera* brood. In contrast, one of the three mites of the K1-1/2 population typical for *A. mellifera* reproduced in *A. cerana* colonies. Drifting of mites between host species thus regularly takes place, with occasionally successful reproduction. These observations are in concordance with recent reports on *Varroa* spp. mites in Thailand (Dietemann et al. 2019). The alternative explanation for the presence of *A.* *mellifera*-like K1-1/2 mites in *A. cerana* colonies is that these mites are natural parasites of the *A. cerana* population screened (rather than drifters). However, this is unlikely given their high degree of differentiation compared to other local K1 mites commonly found in *A.* *cerana* (Table 4).

When drifting is associated with reproduction, opportunities for host shifts occur. Despite high genetic diversity and frequent opportunities for host shifts (Fig. 1) (Dietemann et al. 2019), *Varroa* spp. lineages are only rarely able to reproduce on alternative hosts (Anderson and Trueman 2000; Beaurepaire et al. 2015; Roberts et al. 2015). The traits allowing for reproduction on alternative hosts by *Varroa* spp. still need to be identified in order to evaluate more effectively the risk posed by these mites to *A. mellifera* and potentially to other honeybee species. The K1-1/2 variant infesting *A. cerana* is a good model for this, since it does not reproduce on *A.* *mellifera* and can be compared with the host-shifted K1-1/2.

## Conclusion

The mitochondrial phylogeny at the haplogroup scale from our sampling effort matched well with that of Navajas et al. (2010) and completed our understanding of the radiation within *V. destructor*. At a finer phylogenetic scale, we showed that *V. destructor* is subdivided into several haplogroups and variants, which have apparently undergone distinct evolutionary pathways in line with the geographic mosaic theory of coevolution (Thompson 2005). These pathways have led to populations of low or high genetic diversity. In particular, the K haplotype infesting *A*. *cerana* is more diverse than it has been reported to date (Navajas et al. 2010; Traynor et al. 2020); it is composed of at least 14 variants of two haplogroups, which hints at a high evolutionary potential despite its inbred reproductive system.

Several K1 variants were often sympatric, occasionally infesting the same colonies and likely interbreeding. Diversity was even observed within a single mitochondrial variant. The K1-1/2 populations infesting *A.* *mellifera* and *A. cerana* differed genetically and in their reproductive abilities, which could explain the unidirectional gene flow from the mite lineage infesting the novel host to the mites infesting the original host. This unidirectional flow coupled with an increased differentiation could ultimately lead to the speciation of the invasive lineage. In addition, it may constitute a risk if hybrids disturb the coevolutionary balance with the original host.

Our results, combining behavioral and molecular approaches, allowed for a better understanding of the biological relevance of taxonomic levels in *V. destructor* and of the dispersal, gene flow, and reproductive patterns that affect the micro- and macroevolutionary processes in this parasite. The identification of these processes is pivotal to our ability to prevent the future emergence of infectious diseases, mitigate the detrimental effects of the current damage to *A. mellifera* generated by the invasive *V.* *destructor* lineage, and evaluate the risks this lineage represents to the original host, *A*. *cerana*.

## Authors’ contributions

HZ, VD, FH and PN conceived and designed the research. ZL, SW, LL, PP, and GC collected samples. SW, LL, GC, and ZL performed the molecular work. ZL, SW, LL, VD, and PP performed the experimental infestations. ZL, SW, and VD analyzed the data. VD, ZL, SW, PN, HZ, and PP wrote the manuscript. All authors read and approved the manuscript.

## Supplementary Information

Below is the link to the electronic supplementary material.

Supplementary material 1 (PDF 1462 kb)
