# Supplementary material for: Population genetics and host specificity of Varroa destructor mites infesting eastern and western honeybees
Source: J Pest Sci (2004). 2021 Jan 15;94(4):1487–504. doi: 10.1007/s10340-020-01322-7 (PMC8549952; doi:10.1007/s10340-020-01322-7)
Supplement: Supplementary file 1 — Supplementary material 1 (PDF 1462 kb) [file 10340_2020_1322_MOESM1_ESM.pdf]

Journal of Pest Science **Supplementary Material**

Population genetics and host specificity of *Varroa destructor* mites infesting Eastern and Western honeybees

Zheguang Lin[1,2¶], Shuai Wang[1¶], Peter Neumann[3,4], Gongwen Chen[1], Paul Page[3,4], Li Li[1], Fuliang Hu[1], Huoqing Zheng[1*], Vincent Dietemann[3,5]

[1] College of Animal Sciences, Zhejiang University, Hangzhou, China

[2] College of Animal Science and Technology, Yangzhou University, Yangzhou, China

[3] Swiss Bee Research Center, Agroscope, Bern, Switzerland

[4] Institute of Bee Health, Vetsuisse Faculty, University of Bern, Bern, Switzerland

[5] Department of Ecology and Evolution, University of Lausanne, Lausanne, Switzerland

* Corresponding author: hqzheng@zju.edu.cn

[¶] These authors contributed equally to this work.

Supplementary information includes:

Figure S1

Table S1 – S9

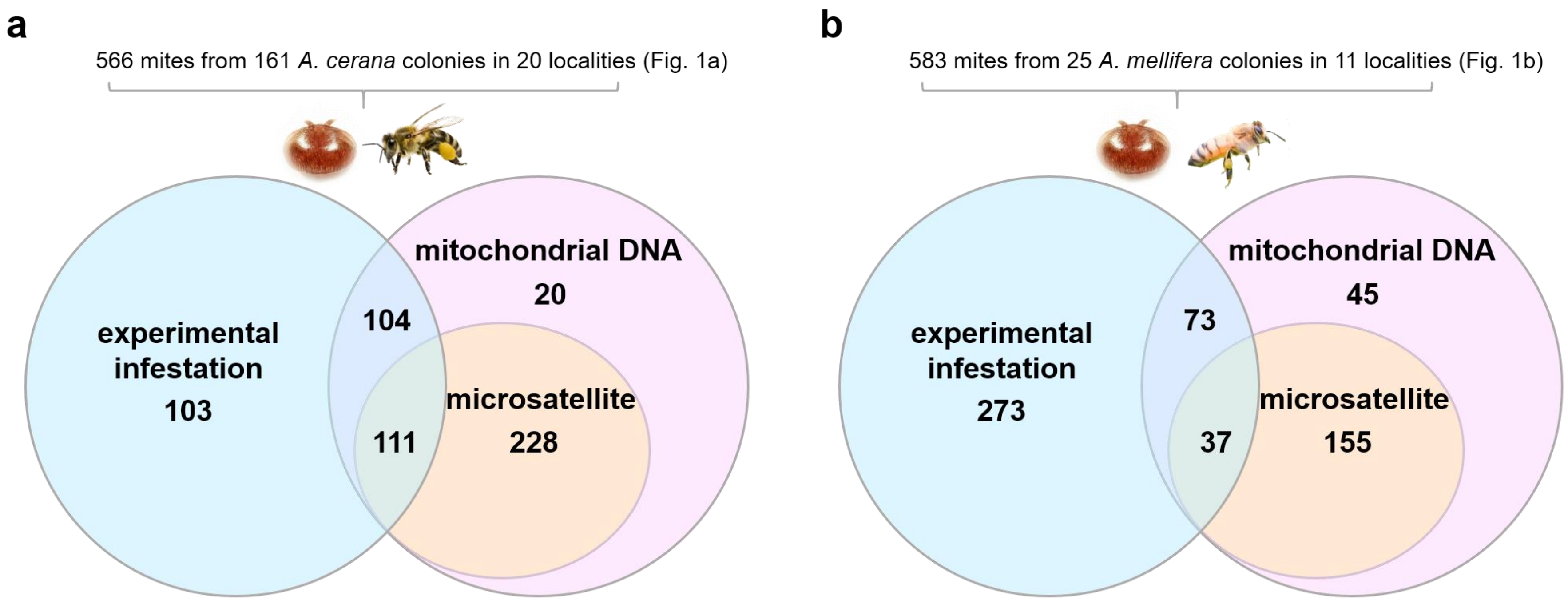

Figure S1. Experimental strategy for the use of *Varroa destructor* mites collected from *Apis cerana* colonies (**a**) and *Apis mellifera* colonies (**b**). The numbers of the mites used in each experiment were shown.

Table S1. Primers for PCR amplification of two mitochondrial DNA fragments of *V. destructor*.

Fragment	Primer Name	Primer sequences (5' – 3')	Size (bp)	Tm (°C)‡	Reference
cox1	Cox1_821_F	GGAGTAGGTACAGGTTGAACGG	821	55	this study
	Cox1_821_R	ACAACCCCAGCAATAATAGCAA			
cox1†	10KbCOIF1	CTTGTAATCATAAGGATATTGGAAC	929	51	Navajas et al. 2010
	6,5KbCOIR	AATACCAGTGGGAACCGC			
cytb	10KbCytbF-1	GCAGCTTTAGTGGATTTACCTAC	985	52	
	10KbCytbPRIM	CTACAGGACACGATCCCAAG			

† This pair of primers was used to distinguish the C1-1 and C1-2 haplotypes since the region distinguishing these variants was not included in the 821-bp *cox1* sequence.

‡ Tm: annealing temperature. Tm values depend on the primer synthesis reports.

Table S2. Information on the microsatellite loci used.

Locus	Size (bp)	Tm (°C)†	Reference
vj275	203–217	58	Evans 2000
vj292	233–235	60	
vj294	169–174	58	
vjL3B2	189–191	54	
VDK00018134-3348 (VDK3348)	330	53	Cornman et al. 2010
VDK00059601-1767 (VDK1767)	127	58	

† Tm: annealing temperature. Tm values depend on the primer synthesis reports.

Table S3. Ouptut of SpadeR package (Chao and Chui 2016) for the estimated *Varroa destructor* variant richness in eastern China.

(1) Basic Data Information

		Variable Value
Sample size	n	463
Number of observed species	D	17
Coverage estimate for entire dataset	C	0.987
CV for entire dataset	CV	1.825
Cut-off point	k	10
Number of observed individuals for rare group	n_rare	16
Number of observed species for rare group	D_rare	10
Estimate of the sample coverage for rare group	C_rare	0.625
Estimate of CV for rare group in ACE	CV_rare	0.258
Estimate of CV1 for rare group in ACE-1	CV1_rare	0.331
Number of observed individuals for abundant group	n_abund	447
Number of observed species for abundant group	D_abund	7

(2) Species Richness Estimators Table

	estimate	s.e.	95% Lower	95% Upper
iChao1 (Chiu et al. 2014)	27.981	10.152	19.353	68.248
ACE (Chao & Lee, 1992)	23.640	6.605	18.307	50.739

Table S4. Sequence alignment of the amplified mtDNA regions of representative individuals. One individual of one haplogroup and variant was selected per sampling location and highlighted with an orange background. Remaining sequences were reported by Navajas et al. (2010) and are retrieved from the GenBank database.

Variable sites — *cox1*

Species	Haplotype	Locality	1350	1362	1401	1404	1425	1542	1566	1584	1587	1602	1644	1677	1716	1731	1737	1746	1773	1791	1902	1959	1983	2127	2130	2136	2244
A. mellifera	K1-1	Seoul(Korea)	T	G	T	A	T	A	T	C	C	T	G	G	G	A	T	G	A	T	A	C	G	C	G	T	A
A. mellifera	K1-1	Tokyo(Japan)	.	.	.	.	.	.	.	.	.	.	.	.	.	.	.	.	.	.	.	.	.	.	.	.	.
A. mellifera	K1-1	Vlasivostok(Russia)	.	.	.	.	.	.	.	.	.	.	.	.	.	.	.	.	.	.	.	.	.	.	.	.	.
A. mellifera	K1-2	Hanoi(Vietnam)	.	.	.	.	.	.	.	.	.	.	.	.	.	.	.	.	.	.	.	.	.	.	.	.	.
A. mellifera	K1-2	Nanchang(China)	.	.	.	.	.	.	.	.	.	.	.	.	.	.	.	.	.	.	.	.	.	.	.	.	.
A. cerana	K1-3	Hunan(China)	.	.	.	.	.	.	.	.	.	.	.	.	.	G	.	.	.	A	.	.	.	.	.	.	.
A. cerana	K1-3	Nanchang(China)	.	.	.	.	.	.	.	.	.	.	.	.	.	G	.	.	.	A	.	.	.	.	.	.	.
A. mellifera	K1-4	Xishuangbanna(China)	.	.	.	.	.	.	.	.	.	.	.	.	.	.	.	.	.	.	.	.	.	.	.	.	G
A. mellifera	K1-1/2	Hangzhou(China)	/	/	/	/	/	.	.	.	.	.	.	.	.	.	.	.	.	.	.	.	.	.	.	.	.
A. cerana	K1-1/2	Hangzhou(China)	/	/	/	/	/	.	.	.	.	.	.	.	.	.	.	.	.	.	.	.	.	.	.	.	.
A. cerana	K1-3	Hangzhou(China)	/	/	/	/	/	.	.	.	.	.	.	.	.	G	.	.	.	A	.	.	.	.	.	.	.
A. mellifera	K1-5	Hangzhou(China)	/	/	/	/	/	.	.	.	.	.	.	.	.	.	.	.	.	.	.	.	.	.	.	.	.
A. cerana	K1-5	Hangzhou(China)	/	/	/	/	/	.	.	.	.	.	.	.	.	.	.	.	.	.	.	.	.	.	.	.	.
A. cerana	K1-6	Chun'an(China)	/	/	/	/	/	.	.	.	.	.	.	.	.	.	.	.	.	.	.	.	.	.	.	.	.
A. cerana	K1-7	Hangzhou(China)	/	/	/	/	/	.	.	.	.	.	.	.	.	.	.	.	.	A	.	.	.	.	.	A	.
A. cerana	K1-8	Nanchang(China)	/	/	/	/	/	.	.	.	.	.	.	.	.	G	.	.	.	A	.	.	.	.	.	.	.
A. cerana	K1-9	Nanchang(China)	/	/	/	/	/	.	.	.	.	.	.	.	.	G	.	.	.	A	.	T	.	.	.	.	.
A. cerana	K1-10	Jinhua(China)	/	/	/	/	/	.	.	T	.	.	.	.	.	G	.	A	.	A	.	.	.	.	.	.	.
A. cerana	K1-11	Fuzhou(China)	/	/	/	/	/	.	.	.	.	.	.	.	.	G		A		A	.	.	.	.	.	.	.
A. cerana	K1-12	Jinhua(China)	/	/	/	/	/	C	.	.	.	.	.	.	.	.	.	.	.	.	.	.	.	.	.	.	.
A. cerana	K1-13	Hangzhou(China)	/	/	/	/	/	.	.	.	.	.	.	.	.	.	.	.	.	A	.	.	.	.	.	.	.
A. cerana	K1-14	Jingmen(China)	/	/	/	/	/	.	.	.	.	.	.	.	.	G	.	.	.	A	.	.	.	T	.	.	.
A. cerana	K1-15	Hangzhou(China)	/	/	/	/	/	C	.	.	.	.	.	.	.	G	.	.	.	A	.	.	.	.	.	.	.
A. cerana	K2-1	Ya'an(China)	/	/	/	/	/	.	.	.	.	.	.	.	.	G	.	.	.	A	.	.	A	.	.	.	G
A. cerana	V1-1	Hanoi(Vietnam)	.	.	C	.	.	.	.	.	.	A	.	.	A	G	C	.	.	A	G	.	A	.	.	.	.
A. cerana	V1-2	Xishuangbanna(China)	.	.	C	.	.	.	.	.	.	A	.	.	A	G	C	.	.	A	G	.	A	.	.	.	.
A. cerana	V1-3	Chiang Mai(Thailand)	.	.	C	.	.	.	.	.	.	A	.	.	A	G	C	.	.	A	G	.	A	.	.	.	.
A. cerana	V1-4	BangChangtay(Thailand)	.	.	C	.	.	.	.	.	.	A	.	.	A	G	.	.	.	A	G	.	A	.	.	.	.
A. cerana	C1-1	Zhuhai(China)	.	.	C	G	.	.	C	T	.	A	.	.	A	G	.	.	.	A	G	T	A	.	A	.	.
A. cerana	C1-2	Zhongshan(China)	.	.	C	.	.	.	C	T	.	A	.	.	A	G	.	.	.	A	G	T	A	.	A	.	.
A. cerana	C1-2	Guangzhou(China)	/	/	C	.	.	.	C	T	.	A	.	.	A	G	.	.	.	A	G	T	A	.	A	.	.
A. cerana	C2-1	Kunming(China)	C	A	C	.	.	.	.	T	T	.	A	A	.	G	.	.	G	A	.	.	A	.	.	.	.
A. cerana	C3-1	Dayao(China)	C	.	C	.	.	.	.	.	T	.	A	A	.	G	.	.	.	A	.	T	A	.	.	.	G
A. cerana	C4-1	Guiyang(China)	/	/	/	/	/	.	.	.	T	.	A	A	.	G	.	.	.	A	.	.	A	.	.	.	G
A. mellifera	J1-1	Taichung(China)	.	.	.	.	C	.	.	.	.	.	.	.	.	G	.	.	.	A	.	.	A	T	.	.	.
A. cerana	J1-2	Tokyo(Japan)	.	.	.	.	C	.	.	.	.	.	.	.	.	G	.	.	.	A	.	.	A	T	.	.	.
A. cerana	J1-3	Machida(Japan)	.	.	.	.	C	.	.	.	.	.	.	.	.	G	.	.	.	A	.	.	A	T	.	.	.
A. cerana	J1-4	Shikoku(Japan)	.	.	.	.	C	.	.	.	.	.	.	.	.	G	.	.	.	A	.	.	A	T	.	.	.
A. mellifera	J1-5	Chiang Mai(Thailand)	.	.	.	.	C	.	.	.	.	.	.	.	.	G	.	.	.	A	.	.	A	T	.	.	.
A. mellifera	J1-6	Tokyo(Japan)	.	.	.	.	C	.	.	.	.	.	.	.	.	G	.	.	.	A	.	.	A	T	.	.	.
A. cerana	J2-1	Antu(China)	/	/	/	/	/	.	.	.	.	.	.	.	.	G	.	.	.	A	.	.	A	.	.	.	.

Variable sites — *cytb*

Species	Haplotype	Locality	9854	9869	9960	9995	10022	10040	10061	10070	10133	10154	10157	10172	10196	10253	10271	10335	10433	10449	10475	10494	10506	10538	10580	10598	10631	10712
A. mellifera	K1-1	Seoul(Korea)	A	C	A	G	T	C	T	T	G	G	C	A	G	A	C	T	C	A	A	T	T	C	A	C	T	T
A. mellifera	K1-1	Tokyo(Japan)	.	.	.	.	.	.	.	.	.	.	.	.	.	.	.	.	.	.	.	.	.	.	.	.	.	.
A. mellifera	K1-1	Vlasivostok(Russia)	.	.	.	.	.	.	.	.	.	.	.	.	.	.	.	.	.	.	.	.	.	.	.	.	.	.
A. mellifera	K1-2	Hanoi(Vietnam)	.	.	.	.	.	.	.	.	.	.	.	.	.	.	.	.	.	.	.	.	.	.	.	.	.	.
A. mellifera	K1-2	Nanchang(China)	.	.	.	.	.	.	.	.	.	.	.	.	.	.	.	.	.	.	.	.	.	.	.	.	.	.
A. cerana	K1-3	Hunan(China)	.	.	.	.	.	.	.	.	.	.	.	.	.	.	.	.	.	.	.	.	.	.	.	.	.	.
A. cerana	K1-3	Nanchang(China)	.	.	.	.	.	.	.	.	.	.	.	.	.	.	.	.	.	.	.	.	.	.	.	.	.	.
A. mellifera	K1-4	Xishuangbanna(China)	.	.	.	.	.	.	.	.	.	.	.	.	.	.	.	.	.	.	.	.	.	.	.	.	.	.
A. mellifera	K1-1/2	Hangzhou(China)	/	/	.	.	.	.	.	.	.	.	.	.	.	.	.	.	.	.	.	.	.	.	.	.	.	.
A. cerana	K1-1/2	Hangzhou(China)	/	/	.	.	.	.	.	.	.	.	.	.	.	.	.	.	.	.	.	.	.	.	.	.	.	.
A. cerana	K1-3	Hangzhou(China)	/	/	.	.	.	.	.	.	.	.	.	.	.	.	.	.	.	.	.	.	.	.	.	.	.	.
A. mellifera	K1-5	Hangzhou(China)	/	/	.	.	.	.	.	.	.	.	.	.	.	.	T	.	.	.	.	.	.	.	.	.	.	.
A. cerana	K1-5	Hangzhou(China)	/	/	.	.	.	.	.	.	.	.	.	.	.	.	T	.	.	.	.	.	.	.	.	.	.	.
A. cerana	K1-6	Chun'an(China)	/	/	.	.	.	.	.	.	.	.	T	.	.	.	.	.	.	.	.	.	.	.	.	.	.	.
A. cerana	K1-7	Hangzhou(China)	/	/	.	.	.	.	.	.	.	.	T	.	.	.	.	.	.	.	.	.	A	.	.	.	.	.
A. cerana	K1-8	Nanchang(China)	/	/	.	.	.	.	.	.	.	.	T	.	.	.	.	.	.	.	.	.	.	.	.	T	.	.
A. cerana	K1-9	Nanchang(China)	/	/	.	.	.	.	.	.	.	.	T	.	.	.	.	.	.	.	.	.	.	.	.	T	.	.
A. cerana	K1-10	Jinhua(China)	/	/	.	.	.	T	.	.	.	.	.	.	.	.	.	.	.	.	.	.	.	.	.	.	.	.
A. cerana	K1-11	Fuzhou(China)	/	/	.	.	.	T	.	.	.	.	.	.	.	.	.	.	.	.	.	.	.	.	.	.	.	.
A. cerana	K1-12	Jinhua(China)	/	/	.	.	.	.	.	.	.	.	.	.	.	.	.	.	.	.	.	.	.	.	.	.	.	.
A. cerana	K1-13	Hangzhou(China)	/	/	.	.	.	.	.	.	.	.	.	T	.	.	.	.	.	.	.	.	A	.	.	.	.	.
A. cerana	K1-14	Jingmen(China)	/	/	.	.	.	.	.	.	.	.	.	.	.	.	.	.	.	.	.	.	.	.	.	.	.	.
A. cerana	K1-15	Hangzhou(China)	/	/	.	.	.	.	.	.	.	.	.	.	.	.	.	.	.	.	.	.	.	.	.	.	.	.
A. cerana	K2-1	Ya'an(China)	/	/	.	.	.	.	.	.	.	A	.	.	.	G	.	.	.	.	.	.	.	.	.	T	.	.
A. cerana	V1-1	Hanoi(Vietnam)	.	.	G	A	.	T	.	.	.	A	.	.	A	.	T	.	.	.	.	.	.	.	.	T	.	C
A. cerana	V1-2	Xishuangbanna(China)	.	T	G	A	A	T	.	.	.	.	.	.	.	.	.	.	.	G	.	C	.	.	.	T	.	C
A. cerana	V1-3	Chiang Mai(Thailand)	.	T	G	.	.	T	.	.	.	A	.	.	.	.	.	.	.	.	.	.	.	.	G	T	.	C
A. cerana	V1-4	BangChangtay(Thailand)	.	T	G	A	A	T	.	.	.	A	.	.	.	.	.	.	.	.	.	C	.	.	.	T	.	C
A. cerana	C1-1	Zhuhai(China)	.	T	G	.	.	T	.	.	.	A	.	.	.	.	.	.	.	.	.	.	.	.	G	T	.	C
A. cerana	C1-2	Zhongshan(China)	.	T	G	.	.	T	.	.	.	A	.	.	.	.	.	.	.	.	.	.	.	.	G	T	.	C
A. cerana	C1-2	Guangzhou(China)	/	T	G	.	.	T	.	.	.	A	.	.	.	.	.	.	.	.	.	.	.	.	G	T	.	C
A. cerana	C2-1	Kunming(China)	.	.	G	.	.	T	G	A	.	A	.	.	.	.	.	C	T	.	.	.	.	.	.	T	.	C
A. cerana	C3-1	Dayao(China)	.	.	G	.	.	T	G	.	.	A	.	.	.	.	.	.	T	.	.	.	.	.	.	T	.	C
A. cerana	C4-1	Guiyang(China)	.	.	G	.	.	T	.	.	.	A	.	.	.	.	.	C	T	.	.	.	.	T	.	T	.	.
A. mellifera	J1-1	Taichung(China)	G	T	.	.	.	T	.	.	.	A	.	.	.	.	.	.	.	.	G	.	.	T	.	T	C	.
A. cerana	J1-2	Tokyo(Japan)	.	T	.	.	.	T	.	.	.	A	.	.	.	.	.	.	.	.	G	.	.	T	.	T	C	.
A. cerana	J1-3	Machida(Japan)	.	T	.	.	.	T	.	.	.	A	.	.	.	.	.	.	.	.	.	.	.	T	.	T	C	.
A. cerana	J1-4	Shikoku(Japan)	.	A	.	.	.	T	.	.	.	A	.	.	.	.	.	.	.	.	.	.	.	T	.	T	C	.
A. mellifera	J1-5	Chiang Mai(Thailand)	.	T	G	A	A	T	.	.	.	A	.	.	.	.	.	.	.	.	.	C	.	.	.	T	.	C
A. mellifera	J1-6	Tokyo(Japan)	.	A	.	.	.	T	.	.	.	A	.	.	.	.	.	.	.	.	G	.	.	T	.	T	C	.
A. cerana	J2-1	Antu(China)	/	T	.	.	.	T	.	.	.	A	.	.	.	.	.	.	.	.	.	.	.	T	.	T	C	.

Table S5. Summary metrics and statistics of the microsatellite markers used, over all haplotypes and variants.

**Locus**	N_{al}	**AR**	H_o	H_e	F_{IS}	G_{ST}	D_{est}	**HWE-*P***
VDK3348	16	27.64	0.13	0.74	0.82***	0.33***	0.76***	***
Vj275	44	44.00	0.54	0.90	0.40***	0.22***	0.72***	***
Vj294	12	17.80	0.14	0.78	0.82***	0.26***	0.66***	***
VDK1767	9	12.94	0.09	0.58	0.85***	0.20**	0.35**	***
Vj292	15	16.79	0.15	0.78	0.81***	0.27***	0.68***	***
VjL3B2	1	1.00	0.00	0.00	N/A	N/A	N/A	N/A
Total						0.30***	0.47***	

N_{al}: Number of alleles; AR: Allelic richness; H_o: Mean observed heterozygosity; H_e: Mean expected heterozygosity; F_{IS}: inbreeding coefficient; G_{ST}: adjusted fixation index; D_{est}: Jost's allelic diversity index; HWE-*P*: Hardy-Weinberg equilibrium *P*-values, *** $P < 0.001$, **$P < 0.01$. N/A: not applicable. See figure 1 for sample size and sampling locations.

Table S6. Microsatellite allele frequencies (in percent) of all *V. destructor* variants for each locus. Red to blue background correspond to decreasing frequency values.

	host species	*A. mellifera*	*A. cerana*																
	variants	K1-1	K1-1	K1-3	K1-5	K1-6	K1-7	K1-8	K1-9	K-10	K1-11	K1-12	K1-13	K1-14	K1-15	K2-1	C1-2	J2-1	C4-1
	N individuals	192	53	40	106	2	21	10	3	2	3	1	1	1	1	1	74	19	1
Loci	alleles																		
VDK3348	296		28.3	1.3	2.4		11.9								50.0				
	305			5.0	4.7		2.4										100.0		
	308		16.0	3.8	10.4		14.3			25.0		100.0				100.0			
	311	1.0	10.4	26.3	5.7		40.5	40.0	33.3		83.3		50.0	100.0	50.0				
	314		13.2	12.5	25.9		4.8												
	317		1.9		0.9						16.7								

320		0.9				2.4						50.0						
323		6.6	12.5	0.5		4.8												
326	99.0	1.9	38.8	34.4	50.0	16.7											2.6	
328				0.5														
329		16.0		1.9	50.0				75.0								5.3	
332				8.5			60.0	66.7									2.6	
335		3.8		4.2		2.4											36.8	
338		0.9															13.2	
341																	21.1	
344																	18.4	
356																		100.0

		K1-1	K1-1	K1-3	K1-5	K1-6	K1-7	K1-8	K1-9	K-10	K1-11	K1-12	K1-13	K1-14	K1-15	K2-1	C1-2	J2-1	C4-1
VJ275	151						3.3												
	153		29.5	10.7	1.5		6.7								50.0			7.7	
	155		26.1	28.6	41.9													34.6	
	157		3.4		14.1													30.8	
	159	0.3	2.3		4.0	50.0	23.3	10.0										3.8	
	161	1.2	3.4	21.4	4.5		6.7	30.0	100.0						50.0		100.0	15.4	
	163		4.5	10.7	1.0	50.0	16.7	55.0											
	165		10.2	28.6	6.1		26.7	5.0										3.8	
	167		1.1		4.5		16.7											3.8	
	169				2.0							100.0							
	171		17.0		3.5														
	173				0.5														

177			0.5	
179			2.0	
181		2.3		
185			0.5	50.0
187			0.5	50.0
195	1.5			
197	8.6		0.5	
199	27.3			
201	25.2			
203	9.2			
205	12.3		9.1	
207	8.3		3.0	
209	1.5			

211	0.6
213	0.6
215	0.3
217	0.3
219	1.2
221	1.2
223	0.3

VJ294	K1-1	K1-1	K1-3	K1-5	K1-6	K1-7	K1-8	K1-9	K-10	K1-11	K1-12	K1-13	K1-14	K1-15	K2-1	C1-2	J2-1	C4-1
164				0.5						33.3	50.0							
166				3.8														
168		5.7		31.6						33.3	50.0		100.0			100.0		50.0
170		6.6	7.5	9.4	50.0	11.9						50.0		100.0				50.0

172	17.2	22.6	36.3	15.1	50.0	11.9	20.0			16.7							
174	82.8	11.3	15.0	9.4		9.5	55.0	83.3	100.0	16.7							
175				0.9													
176		3.8	5.0	4.2		47.6	25.0	16.7				50.0					7.9	
178		14.2													100.0		15.8	
180		35.8	35.0	2.4		19.0											21.1	
182			1.3	22.6													18.4	
184																	15.8	
186																	21.1	

VDK1767	K1-1	K1-1	K1-3	K1-5	K1-6	K1-7	K1-8	K1-9	K-10	K1-11	K1-12	K1-13	K1-14	K1-15	K2-1	C1-2	J2-1	C4-1
107		1.9																
113				3.8														

116							15.0											
119			5.0	17.0		4.8											7.9	
122		4.7	13.8	3.3		9.5	10.0					50.0					47.4	
125		3.8	10.0	19.3		42.9	55.0	16.7	100.0				100.0	50.0			18.4	
128	100.0	28.3	31.3	45.8	25.0	16.7	20.0	83.3		50.0	100.0	50.0			100.0	60.8	26.3	100.0
131		44.3	40.0	10.8	75.0	26.2				50.0								
134		17.0												50.0		39.2		

VJ292	K1-1	K1-1	K1-3	K1-5	K1-6	K1-7	K1-8	K1-9	K-10	K1-11	K1-12	K1-13	K1-14	K1-15	K2-1	C1-2	J2-1	C4-1
217											50.0						2.6	
219		7.5	2.5	27.4		23.8	10.0								100.0	100.0	89.5	50.0
221		1.9	2.5	0.5		2.4				33.3								50.0
223		28.3	40.0	11.8	50.0	9.5	65.0	100.0	50.0	33.3				50.0				

225		1.9		9.0		2.4		50.0						7.9
227					25.0				33.3					
229		3.8	7.5	5.2		33.3	20.0			50.0			50.0	
231		2.8	2.5	0.9		16.7	5.0							
232						2.4								
233	100.0	3.8		0.9	25.0									
235		13.2		22.2								50.0		
237		26.4	22.5	0.9		2.4					50.0	50.0		
241				21.2										
243		1.9	20.0			7.1					50.0			
244		0.9												
251		7.5	2.5											

VJL3B2	K1-1	K1-1	K1-3	K1-5	K1-6	K1-7	K1-8	K1-9	K-10	K1-11	K1-12	K1-13	K1-14	K1-15	K2-1	C1-2	J2-1	C4-1
189	100.0	100.0	100.0	100.0	100.0	100.0	100.0	100.0	100.0	100.0	100.0	100.0	100.0	100.0	100.0	100.0	100.0	100.0

Table S7. Multilocus genotype identities and frequencies for the variants sampled in eastern China. Colors from red to blue indicate decreasing numbers of individuals with a particular genotype.

host species	*A. mellifera*	*A. cerana*																
haplogroup		K1													K2	J2	C1	C4
variant	1/2	1/2	3	5	6	7	8	9	10	11	12	13	14	15	1	1	2	1
MLG #																		
183	154	-	-	-	-	-	-	-	-	-	-	-	-	-	-	-	-	-
181	30	-	-	-	-	-	-	-	-	-	-	-	-	-	-	-	-	-
182	6	-	-	-	-	-	-	-	-	-	-	-	-	-	-	-	-	-
139	2	-	-	-	-	-	-	-	-	-	-	-	-	-	-	-	-	-
104	-	7	-	-	-	-	-	-	-	-	-	-	-	-	-	-	-	-
36	-	3	-	-	-	-	-	-	-	-	-	-	-	-	-	-	-	-

23	-	2	-	-	-	-	-	-	-	-	-	-	-	-	-	-	-	-
66	-	2	-	-	-	-	-	-	-	-	-	-	-	-	-	-	-	-
39	-	2	-	-	-	-	-	-	-	-	-	-	-	-	-	-	-	-
76	-	2	-	-	-	-	-	-	-	-	-	-	-	-	-	-	-	-
88	-	2	-	-	-	-	-	-	-	-	-	-	-	-	1	-	-	-
103	-	2	-	-	-	-	-	-	-	-	-	-	-	-	-	-	-	-
116	-	2	-	-	-	-	-	-	-	-	-	-	-	-	-	-	-	-
130	-	2	-	-	-	-	-	-	-	-	-	-	-	-	-	-	-	-
38	-	1	-	-	-	-	-	-	-	-	-	-	-	-	-	-	-	-
21	-	1	-	-	-	-	-	-	-	-	-	-	-	-	-	-	-	-
25	-	1	-	-	-	-	-	-	-	-	-	-	-	-	-	-	-	-
30	-	1	-	-	-	-	-	-	-	-	-	-	-	-	-	-	-	-
31	-	1	-	-	-	-	-	-	-	-	-	-	-	-	-	-	-	-

32	-	1	-	-	-	-	-	-	-	-	-	-	-	-	-	-	-	-
50	-	1	-	-	-	-	-	-	-	-	-	-	-	-	-	-	-	-
65	-	1	-	-	-	-	-	-	-	-	-	-	-	-	-	-	-	-
75	-	1	-	-	-	-	-	-	-	-	-	-	-	-	-	-	-	-
81	-	1	-	-	-	-	-	-	-	-	-	-	-	-	-	-	-	-
85	-	1	-	-	-	-	-	-	-	-	-	-	-	-	-	-	-	-
86	-	1	-	-	-	-	-	-	-	-	-	-	-	-	-	-	-	-
87	-	1	-	-	-	-	-	-	-	-	-	-	-	-	-	-	-	-
93	-	1	-	-	-	-	-	-	-	-	-	-	-	-	-	-	-	-
95	-	1	-	-	-	-	-	-	-	-	-	-	-	-	-	-	-	-
97	-	1	-	-	-	-	-	-	-	-	-	-	-	-	-	-	-	-
98	-	1	-	-	-	-	-	-	-	-	-	-	-	-	-	-	-	-
99	-	1	-	-	-	-	-	-	-	-	-	-	-	-	-	-	-	-

100	-	1	-	-	-	-	-	-	-	-	-	-	-	-	-	-	-	-
102	-	1	-	-	-	-	-	-	-	-	-	-	-	-	-	-	-	-
107	-	1	-	-	-	-	-	-	-	-	-	-	-	-	-	-	-	-
108	-	1	-	-	-	-	-	-	-	-	-	-	-	-	-	-	-	-
114	-	1	-	-	-	-	-	-	-	-	-	-	-	-	-	-	-	-
115	-	1	-	-	-	-	-	-	-	-	-	-	-	-	-	-	-	-
117	-	1	-	-	-	-	-	-	-	-	-	-	-	-	-	-	-	-
136	-	1	-	-	-	-	-	-	-	-	-	-	-	-	-	-	-	-
162	-	1	-	-	-	-	-	-	-	-	-	-	-	-	-	-	-	-
174	-	-	6	-	-	-	-	-	-	-	-	-	-	-	-	-	-	-
67	-	-	4	-	-	-	-	-	-	-	-	-	-	-	-	-	-	-
133	-	-	4	-	-	-	-	-	-	-	-	-	-	-	-	-	-	-
24	-	-	3	-	-	1	-	-	-	-	-	-	-	-	-	-	-	-

22	-	-	2	-	-	-	-	-	-	-	-	-	-	-	-	-	-	-
134	-	-	2	-	-	-	-	-	-	-	-	-	-	-	-	-	-	-
129	-	-	2	-	-	-	-	-	-	-	-	-	-	-	-	-	-	-
147	-	-	2	-	-	-	-	-	-	-	-	-	-	-	-	-	-	-
156	-	-	2	-	-	-	-	-	-	-	-	-	-	-	-	-	-	-
157	-	-	2	-	-	-	-	-	-	-	-	-	-	-	-	-	-	-
159	-	-	2	-	-	-	-	-	-	-	-	-	-	-	-	-	-	-
163	-	-	2	-	-	-	-	-	-	-	-	-	-	-	-	-	-	-
160	-	-	1	-	-	-	-	-	-	-	-	-	-	-	-	-	-	-
148	-	-	1	-	-	-	-	-	-	-	-	-	-	-	-	-	-	-
68	-	-	1	-	-	-	-	-	-	-	-	-	-	-	-	-	-	-
90	-	-	1	-	-	-	-	-	-	-	-	-	-	-	-	-	-	-
94	-	-	1	-	-	-	-	-	-	-	-	-	-	-	-	-	-	-

152	-	-	1	-	-	-	-	-	-	-	-	-	-	-	-	-	-	-
155	-	-	1	-	-	-	-	-	-	-	-	-	-	-	-	-	-	-
58	-	-	-	9	-	-	-	-	-	-	-	-	-	-	-	-	-	-
171	-	-	-	6	-	-	-	-	-	-	-	-	-	-	-	-	-	-
143	-	-	-	5	-	-	-	-	-	-	-	-	-	-	-	-	-	-
142	-	-	-	4	-	-	-	-	-	-	-	-	-	-	-	-	-	-
69	-	-	-	4	-	-	-	-	-	-	-	-	-	-	-	-	-	-
170	-	-	-	4	-	-	-	-	-	-	-	-	-	-	-	-	-	-
92	-	-	-	3	-	-	-	-	-	-	-	-	-	-	-	-	-	-
124	-	-	-	3	-	-	-	-	-	-	-	-	-	-	-	-	-	-
180	-	-	-	3	-	-	-	-	-	-	-	-	-	-	-	-	-	-
20	-	-	-	2	-	-	-	-	-	-	-	-	-	-	-	-	-	-
73	-	-	-	2	-	-	-	-	-	-	-	-	-	-	-	-	-	-

84	-	-	-	2	-	-	-	-	-	-	-	-	-	-	-	-	-	-
119	-	-	-	2	-	-	-	-	-	-	-	-	-	-	-	-	-	-
144	-	-	-	2	-	-	-	-	-	-	-	-	-	-	-	-	-	-
164	-	-	-	2	-	-	-	-	-	-	-	-	-	-	-	-	-	-
167	-	-	-	2	-	-	-	-	-	-	-	-	-	-	-	-	-	-
168	-	-	-	2	-	-	-	-	-	-	-	-	-	-	-	-	-	-
9	-	-	-	1	-	-	-	-	-	-	-	-	-	-	-	-	-	-
10	-	-	-	1	-	-	-	-	-	-	-	-	-	-	-	-	-	-
11	-	-	-	1	-	-	-	-	-	-	-	-	-	-	-	-	-	-
17	-	-	-	1	-	-	-	-	-	-	-	-	-	-	-	-	-	-
26	-	-	-	1	-	-	-	-	-	-	-	-	-	-	-	-	-	-
33	-	-	-	1	-	-	-	-	-	-	-	-	-	-	-	-	-	-
35	-	-	-	1	-	-	-	-	-	-	-	-	-	-	-	-	-	-

45	-	-	-	1	-	-	-	-	-	-	-	-	-	-	-	-	-	-
51	-	-	-	1	-	-	-	-	-	-	-	-	-	-	-	-	-	-
52	-	-	-	1	-	-	-	-	-	-	-	-	-	-	-	-	-	-
53	-	-	-	1	-	-	-	-	-	-	-	-	-	-	-	-	-	-
54	-	-	-	1	-	-	-	-	-	-	-	-	-	-	-	-	-	-
55	-	-	-	1	-	-	-	-	-	-	-	-	-	-	-	-	-	-
56	-	-	-	1	-	-	-	-	-	-	-	-	-	-	-	-	-	-
57	-	-	-	1	-	-	-	-	-	-	-	-	-	-	-	-	-	-
59	-	-	-	1	-	-	-	-	-	-	-	-	-	-	-	-	-	-
60	-	-	-	1	-	-	-	-	-	-	-	-	-	-	-	-	-	-
61	-	-	-	1	-	-	-	-	-	-	-	-	-	-	-	-	-	-
62	-	-	-	1	-	-	-	-	-	-	-	-	-	-	-	-	-	-
63	-	-	-	1	-	-	-	-	-	-	-	-	-	-	-	-	-	-

70	-	-	-	1	-	-	-	-	-	-	-	-	-	-	-	-	-	-
71	-	-	-	1	-	-	-	-	-	-	-	-	-	-	-	-	-	-
72	-	-	-	1	-	-	-	-	-	-	-	-	-	-	-	-	-	-
74	-	-	-	1	-	-	-	-	-	-	-	-	-	-	-	-	-	-
77	-	-	-	1	-	-	-	-	-	-	-	-	-	-	-	-	-	-
79	-	-	-	1	-	-	-	-	-	-	-	-	-	-	-	-	-	-
80	-	-	-	1	-	-	-	-	-	-	-	-	-	-	-	-	-	-
82	-	-	-	1	-	-	-	-	-	-	-	-	-	-	-	-	-	-
91	-	-	-	1	-	-	-	-	-	-	-	-	-	-	-	-	-	-
96	-	-	-	1	-	-	-	-	-	-	-	-	-	-	-	-	-	-
105	-	-	-	1	-	-	-	-	-	-	-	-	-	-	-	-	-	-
106	-	-	-	1	-	-	-	-	-	-	-	-	-	-	-	-	-	-
118	-	-	-	1	-	-	-	-	-	-	-	-	-	-	-	-	-	-

132	-	-	-	1	-	-	-	-	-	-	-	-	-	-	-	-	-	-
141	-	-	-	1	-	-	-	-	-	-	-	-	-	-	-	-	-	-
145	-	-	-	1	-	-	-	-	-	-	-	-	-	-	-	-	-	-
146	-	-	-	1	-	-	-	-	-	-	-	-	-	-	-	-	-	-
149	-	-	-	1	-	-	-	-	-	-	-	-	-	-	-	-	-	-
151	-	-	-	1	-	-	-	-	-	-	-	-	-	-	-	-	-	-
154	-	-	-	1	-	-	-	-	-	-	-	-	-	-	-	-	-	-
158	-	-	-	1	-	-	-	-	-	-	-	-	-	-	-	-	-	-
161	-	-	-	1	-	-	-	-	-	-	-	-	-	-	-	-	-	-
165	-	-	-	1	-	-	-	-	-	-	-	-	-	-	-	-	-	-
166	-	-	-	1	-	-	-	-	-	-	-	-	-	-	-	-	-	-
169	-	-	-	1	-	-	-	-	-	-	-	-	-	-	-	-	-	-
172	-	-	-	1	-	-	-	-	-	-	-	-	-	-	-	-	-	-

173	-	-	-	1	-	-	-	-	-	-	-	-	-	-	-	-	-	-
175	-	-	-	1	-	-	-	-	-	-	-	-	-	-	-	-	-	-
179	-	-	-	1	-	-	-	-	-	-	-	-	-	-	-	-	-	-
34	-	-	-	-	1	-	-	-	-	-	-	-	-	-	-	-	-	-
178	-	-	-	-	1	-	-	-	-	-	-	-	-	-	-	-	-	-
89	-	-	-	-	-	3	-	-	-	-	-	-	-	-	-	-	-	-
125	-	-	-	-	-	3	-	-	-	-	-	-	-	-	-	-	-	-
121	-	-	-	-	-	2	-	-	-	-	-	-	-	-	-	-	-	-
153	-	-	-	-	-	2	-	-	-	-	-	-	-	-	-	-	-	-
122	-	-	-	-	-	1	-	-	-	-	-	-	-	-	-	-	-	-
101	-	-	-	-	-	1	-	-	-	-	-	-	-	-	-	-	-	-
111	-	-	-	-	-	1	-	-	-	-	-	-	-	-	-	-	-	-
64	-	-	-	-	-	1	-	-	-	-	-	-	-	-	-	-	-	-

113	-	-	-	-	-	1	-	-	-	-	-	1	-	-	-	-	-	-
131	-	-	-	-	-	1	-	-	-	-	-	-	-	-	-	-	-	-
138	-	-	-	-	-	1	-	-	-	-	-	-	-	-	-	-	-	-
150	-	-	-	-	-	1	-	-	-	-	-	-	-	-	-	-	-	-
176	-	-	-	-	-	1	-	-	-	-	-	-	-	-	-	-	-	-
177	-	-	-	-	-	1	-	-	-	-	-	-	-	-	-	-	-	-
15	-	-	-	-	-	-	2	-	-	-	-	-	-	-	-	-	-	-
135	-	-	-	-	-	-	2	-	-	-	-	-	-	-	-	-	-	-
8	-	-	-	-	-	-	1	-	-	-	-	-	-	-	-	-	-	-
12	-	-	-	-	-	-	1	-	-	-	-	-	-	-	-	-	-	-
14	-	-	-	-	-	-	1	-	-	-	-	-	-	-	-	-	-	-
109	-	-	-	-	-	-	1	-	-	-	-	-	-	-	-	-	-	-
110	-	-	-	-	-	-	1	-	-	-	-	-	-	-	-	-	-	-

126	-	-	-	-	-	-	1	-	-	-	-	-	-	-	-	-	-	-
13	-	-	-	-	-	-	-	1	-	-	-	-	-	-	-	-	-	-
16	-	-	-	-	-	-	-	1	-	-	-	-	-	-	-	-	-	-
137	-	-	-	-	-	-	-	1	-	-	-	-	-	-	-	-	-	-
40	-	-	-	-	-	-	-	-	1	-	-	-	-	-	-	-	-	-
78	-	-	-	-	-	-	-	-	1	-	-	-	-	-	-	-	-	-
112	-	-	-	-	-	-	-	-	-	1	-	-	-	-	-	-	-	-
123	-	-	-	-	-	-	-	-	-	1	-	-	-	-	-	-	-	-
128	-	-	-	-	-	-	-	-	-	1	-	-	-	-	-	-	-	-
83	-	-	-	-	-	-	-	-	-	-	1	-	-	-	-	-	-	-
127	-	-	-	-	-	-	-	-	-	-	-	-	1	-	-	-	-	-
120	-	-	-	-	-	-	-	-	-	-	-	-	-	1	-	-	-	-
18	-	-	-	-	-	-	-	-	-	-	-	-	-	-	-	29	-	-

19	-	-	-	-	-	-	-	-	-	-	-	-	-	-	-	45	-	-
2	-	-	-	-	-	-	-	-	-	-	-	-	-	-	-	-	1	-
3	-	-	-	-	-	-	-	-	-	-	-	-	-	-	-	-	1	-
4	-	-	-	-	-	-	-	-	-	-	-	-	-	-	-	-	1	-
5	-	-	-	-	-	-	-	-	-	-	-	-	-	-	-	-	1	-
6	-	-	-	-	-	-	-	-	-	-	-	-	-	-	-	-	1	-
7	-	-	-	-	-	-	-	-	-	-	-	-	-	-	-	-	1	-
27	-	-	-	-	-	-	-	-	-	-	-	-	-	-	-	-	1	-
28	-	-	-	-	-	-	-	-	-	-	-	-	-	-	-	-	1	-
29	-	-	-	-	-	-	-	-	-	-	-	-	-	-	-	-	1	-
37	-	-	-	-	-	-	-	-	-	-	-	-	-	-	-	-	1	-
41	-	-	-	-	-	-	-	-	-	-	-	-	-	-	-	-	1	-
42	-	-	-	-	-	-	-	-	-	-	-	-	-	-	-	-	1	-

43	-	-	-	-	-	-	-	-	-	-	-	-	-	-	-	-	1	-
44	-	-	-	-	-	-	-	-	-	-	-	-	-	-	-	-	1	-
46	-	-	-	-	-	-	-	-	-	-	-	-	-	-	-	-	1	-
47	-	-	-	-	-	-	-	-	-	-	-	-	-	-	-	-	1	-
48	-	-	-	-	-	-	-	-	-	-	-	-	-	-	-	-	1	-
49	-	-	-	-	-	-	-	-	-	-	-	-	-	-	-	-	1	-
140	-	-	-	-	-	-	-	-	-	-	-	-	-	-	-	-	1	-
1	-	-	-	-	-	-	-	-	-	-	-	-	-	-	-	-	-	1

Table S8. Ouptut of SpadeR package (Chao and Chui 2016) for the estimated *Varroa destructor* variant richness in the samples used for reproduction abilities evaluation.

(1) Basic Data Information

		Variable Value
Sample size	n	174
Number of observed species	D	6
Coverage estimate for entire dataset	C	0.989
CV for entire dataset	CV	1.008
Cut-off point	k	10
Number of observed individuals for rare group	n_rare	2
Number of observed species for rare group	D_rare	2
Estimate of the sample coverage for rare group	C_rare	0
Estimate of CV for rare group in ACE	CV_rare	0
Estimate of CV1 for rare group in ACE-1	CV1_rare	0
Number of observed individuals for abundant group	n_abund	172
Number of observed species for abundant group	D_abund	4

(2) Species Richness Estimators Table

	estimate	s.e.	95% Lower	95% Upper
iChao1 (Chiu et al. 2014)	6.994	2.208	6.073	19.594
ACE (Chao & Lee, 1992)	6.994	2.208	6.073	19.594

Table S9. Summary statistics of the zero-inflated Bernoulli and binomial models applied to reproductive success of mites in the cross-fostering experiment. H designate hosts experimentally infested and M (for mites) follows the name of the original host species in which the mite was collected. In the Bernoulli models, cases with no reproduction were coded as 0 and those with reproduction as 1. Estimated mean probabilities of mites achieving reproductive success are thus calculated. In the binomial model, the estimated mean probabilities of successful reproduction are calculated.

model including all samples experimentally infested		
factors retained in best model	**host_species_origin + host_species_infested**	**host_species_infested**

bernoulli part of the model

factor / group

host_species_infested

contrast	estimate	SE	df	z.ratio	p.value
A_ceranaH - A_melliferaH	0.18	0.09	NA	-2.00	0.046

binomial part of the model

factor / group

host_species_infested

contrast	estimate	SE	df	z.ratio	p.value
A_ceranaH - A_melliferaH	-0.17	0.05	NA	-3.52	0.0004

	probability	SE	df	asymp.LCL	asymp.UCL
A_ceranaH	0.64	0.07	NA	0.23	0.50
A_melliferaH	0.56	0.06	NA	0.43	0.66

host species_origin

contrast	estimate	SE	df	z.ratio	p.value
A_ceranaM - A_melliferaM	-0.74	0.09	NA	8.10	<0.0001***

	probability	SE	df	asymp.LCL	asymp.UCL

	probability	SE	df	asymp.LCL	asymp.UCL
A_ceranaH	0.29	0.03	NA	0.23	0.36
A_melliferaH	0.46	0.03	NA	0.39	0.52

A_ceranaM	0.18	0.08	NA	0.68	0.97
A_melliferaM	0.92	0.05	NA	-0.02	0.19
**host_species_infested: host species_origin**					
contrast	estimate	SE	df	z.ratio	p.value
A_ceranaH,A_ceranaM - A_melliferaH,A_ceranaM	0.24	0.13	NA	-1.84	0.26
A_ceranaH,A_ceranaM - A_ceranaH,A_melliferaM	-0.68	0.12	NA	5.51	<0.0001 ***
A_melliferaH,A_ceranaM - A_melliferaH,A_melliferaM	-0.80	0.10	NA	8.038	<0.0001 ***

		probability	SE	df	asymp.LCL	asymp.UCL
A_ceranaH,A_melliferaM		-		NA		
A_melliferaH,A_melliferaM		0.12	0.09		-1.39	0.51

		probability	SE	df	asymp.LCL	asymp.UCL
A_ceranaH	A_ceranaM	0.30	0.13	NA	0.50	0.96
A_melliferaH	A_ceranaM	0.06	0.06	NA	0.83	1.06
A_ceranaH	A_melliferaM	0.98	0.03	NA	-0.03	0.08
A_melliferaH	A_melliferaM	0.86	0.09	NA	-0.04	0.33

model including only genotyped individuals

**host_sex**					
contrast	estimate	SE	df	z.ratio	p.value
drone - worker	0.22	0.10	NA	-2.18	0.03
	probability	SE	df	asymp.LCL	asymp.UCL
drone	0.33	0.08	NA	0.50	0.83
worker	0.11	0.06	NA	0.78	1.00

**host_sex**					
contrast	estimate	SE	df	z.ratio	p.value
drone - worker	0.16	0.11	NA	1.51	0.13
	probability	SE	df	asymp.LCL	asymp.UCL
drone	0.49	0.09	NA	0.31	0.67
worker	0.33	0.06	NA	0.22	0.43

References

Cornman RS, Schatz MC, Johnston JS, Chen YP, Pettis J, Hunt G, ... Evans JD (2010) Genomic survey of the ectoparasitic mite *Varroa destructor*, a major pest of the honey bee *Apis mellifera*. BMC Genomics 11(1):602. doi: 10.1186/1471-2164-11-602

Evans JD (2000) Microsatellite loci in the honey bee parasitic mite *Varroa jacobsoni*. Molecular Ecology 9(9):1436-1438. doi: 10.1046/j.1365-294x.2000.00998-3.x

Navajas M, Anderson DL, de Guzman LI, Huang Z, Clement J, Zhou T, Le Conte Y (2010) New Asian types of *Varroa destructor*: a potential new threat for world apiculture. Apidologie 41(2):181-193. doi: 10.1051/apido/2009068
